# Meroterpenoids: A Comprehensive Update Insight on Structural Diversity and Biology †

**DOI:** 10.3390/biom11070957

**Published:** 2021-06-29

**Authors:** Mamona Nazir, Muhammad Saleem, Muhammad Imran Tousif, Muhammad Aijaz Anwar, Frank Surup, Iftikhar Ali, Daijie Wang, Nilufar Z. Mamadalieva, Elham Alshammari, Mohamed L. Ashour, Ahmed M. Ashour, Ishtiaq Ahmed, Elizbit, Ivan R. Green, Hidayat Hussain

**Affiliations:** 1 Department of Chemistry, Government Sadiq College Women University Bahawalpur, Bahawalpur 63100, Pakistan; 2 Institute of Chemistry, Baghdad-ul-Jadeed Campus, The Islamia University of Bahawalpur, Bahawalpur 63100, Pakistan; m.saleem@iub.edu.pk; 3 Department of Chemistry, DG Khan Campus, University of Education Lahore, Dera Ghazi Khan 32200, Pakistan; imran.tousif@ue.edu.pk; 4 Pharmaceutical Research Division, PCSIR Laboratories Complex Karachi, Karachi 75280, Pakistan; maanwarpk@yahoo.com; 5 Microbial Drugs, Helmholtz Centre for Infection Research, 38124 Braunschweig, Germany; Frank.Surup@helmholtz-hzi.de; 6 School of Pharmaceutical Sciences and Key Laboratory for Applied Technology of Sophisticated Analytical Instruments of Shandong Province, Shandong Analysis and Test Center, School of Pharmaceutical Sciences, Qilu University of Technology (Shandong Academy of Sciences), Jinan 250014, China; iftikhar.ali@kiu.edu.pk (I.A.); wangdaijie@qlu.edu.cn (D.W.); 7 Department of Chemistry, Karakoram International University, Gilgit 15100, Pakistan; 8 Department of Bioorganic Chemistry, Leibniz Institute of Plant Biochemistry, Weinberg 3, D-06120 Halle, Germany; nmamadalieva@yahoo.com; 9 Institute of the Chemistry of Plant Substances, Uzbekistan Academy of Sciences, Mirzo Ulugbek Str 77, Tashkent 100170, Uzbekistan; 10 Department of Pharmacy Practice, College of Pharmacy, Princess Nourah Bint Abdulrahman University, Riyadh 11671, Saudi Arabia; ejalshammari@pnu.edu.sa; 11 Department of Pharmaceutical Sciences, Pharmacy Program, Batterjee Medical College, Jeddah 21442, Saudi Arabia; mohamed.ashour@bmc.edu.sa or; 12 Department of Pharmacognosy, Faculty of Pharmacy, Ain Shams University, Cairo 11566, Egypt; 13 Department of Pharmacology and Toxicology, College of Pharmacy, Umm Al-Qura University, P.O. Box 13578, Makkah 21955, Saudi Arabia; amashour@uqu.edu.sa; 14 Department of Chemical Engineering and Biotechnology, University of Cambridge, Cambridge CB2 1TN, UK; 15 Department of Materials Engineering, National University of Sciences and Technology (NUST) H12, Islamabad 44000, Pakistan; malkaalizachem@gmail.com; 16 Department of Chemistry and Polymer Science, University of Stellenbosch, Private Bag X1, Matieland, Stellenbosch 7600, South Africa; irg@sun.ac.za

**Keywords:** meroterpenoids, structural diversity, antidiabetic, anticancer, antioxidant, anti-inflammatory

## Abstract

Meroterpenoids are secondary metabolites formed due to mixed biosynthetic pathways which are produced in part from a terpenoid co-substrate. These mixed biosynthetically hybrid compounds are widely produced by bacteria, algae, plants, and animals. Notably amazing chemical diversity is generated among meroterpenoids via a combination of terpenoid scaffolds with polyketides, alkaloids, phenols, and amino acids. This review deals with the isolation, chemical diversity, and biological effects of 452 new meroterpenoids reported from natural sources from January 2016 to December 2020. Most of the meroterpenoids possess antimicrobial, cytotoxic, antioxidant, anti-inflammatory, antiviral, enzyme inhibitory, and immunosupressive effects.

## 1. Introduction

Natural products and their analogs in today’s age play a crucial role in the development of novel drugs because of their tremendous structural diversity [1,2,3,4,5]. It has been reported that out of the 877 New Chemical Entities (NCEs) established between 1981 and 2002, ca. 49% arose from natural products, or, synthetic molecules based on natural-product-pharmacophores [6,7]. However, pharmaceutical research into natural secondary metabolites has declined in the last two decades because of the difficulty in isolating compounds with skeletally novel frameworks from natural resources rather than from combinatorial synthetic protocols [7,8,9]. 

The term “meroterpenoid” was first used by Cornforth [10] in 1986 to describe natural products bearing a mixed terpenoid biogenesis. The prefix “*mero*-” is derived from the Greek word “*merus*” which means “fragment, or part, or partial” [11,12,13]. Meroterpenoids are thus a class of natural products derived from hybrid polyketide or non-polyketide and terpenoid biosynthesis. The unusual enzyme reactions responsible for connectivities among their structures, and their unique ring cores create a most interesting chemical diversity among meroterpenoids [11,14,15]. Interestingly, meroterpenoids are mostly reported from fungi and marine organisms while only a limited number of meroterpenoids were obtained from plants [9]. 

Ubiquinone-10, α-tocopherol and the anticancer drugs, viz., vinblastine and vincristine are included in the meroterpenoid classification [12,13]. Due to the interesting chemical diversity, meroterpenoids illustrated an impressive range of biological effects, viz., antioxidant [16,17,18], anti-inflammatory [19,20,21,22], anti-atherosclerotic [23], anti-adipogenic [24], anti-diabetic [25], anti-carcinogenic [26,27], and neuroprotective [28] activities. In 2009, Geris and Simpson [12] published a review on fungal meroterpenoids. Peng and Qiu [13] reported on 135 meroterpenoids which were all obtained from the fungal genus *Ganoderma* sp. Quite recently, another four reviews appeared: (i) El-Demerdash et al. [29] summarized 320 meroterpenoids from marine fungi covering from 2009 to 2019. (ii) Zhao et al. [30] focused on terrestrial and marine-derived fungal 709 meroterpenoids (covered 2009–2020). (iii) Gozari et al. [31] reviewed only 190 meroterpenoids from bacteria and fungi that showed significant preclinical effects. (iv) Jiang et al. [32] summarized the described 1585 fungal meroterpenoids in 2009–2019. 

Our review article describes the systematic and complete summary of potentially bioactive meroterpenoids from all natural sources except fungal meroterpenoids during the last five years 2016–2020 (January 2016 to December 2020). In point of fact, an amazing 452 new meroterpenoids were discovered during this period, which were mostly tested for their various biological activities. 

## 2. Meroterpenoid Classification

The structures of meroterpenoids are exceptionally diverse and complex which is why classifications of these compounds are not easy. Meroterpenoids were often classified in two ways. The first way is to classify these compounds as polyketide- and non-polyketide-terpenoids previously described by Geris and Simpson [12]. The second way is to classify these compounds based on a common scaffold, common natural product skeleton, or heterocyclic ring system, viz., phloroglucinol, syncarpic acid, sesquiterpene, phthalide, benzofuran and phenylfuran [11,12]. We followed the second method.

## 3. Phloroglucinol-Based Meroterpenoids

**Figure 1:**
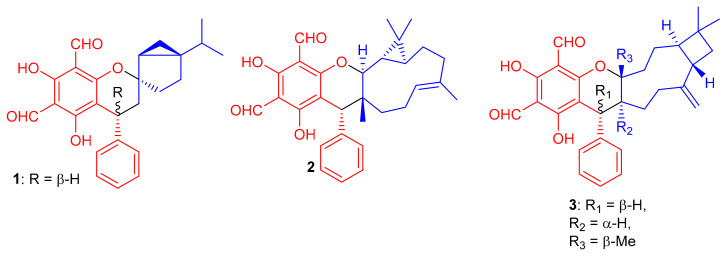
Structures of phloroglucinol-based meroterpenoids **1**–**3**.

Among other anticancer meroterpenoids, diformylphloroglucinol-derived meroterpenoids from *Psidium guajava* L. were identified by spectroscopic analyses and ECD calculations as psiguajavadials A (**1**) and B (**2**), and guajadial (**3**) (Figure 1) [33]. All these metabolites showed antitumor activity against HCT116, Huh7, DU145, CCRF-CEM, and A549 cells. (Table 1). Compounds **1** and **2** displayed dose-dependent inhibition of Top1 activity [33]. Guajadial (**3**), inhibited endothelial cell proliferation and migration as well as suppressing tumor growth in human NSCLC (A549 and H1650 cells) and xenograft mouse models. This potential has been reported as being a significant antineoplasmic activity of **3**. The Western blotting method to study the underlying mechanisms of VEGF receptor (VEGFR)2-mediated revealed that compound **3** inhibited A549 (IC_50_ = 3.58 μM) proliferation via blocking the Ras/MAPK pathway [34]; this activity of **3** is higher than the potential of cisplatin (IC_50_ value of 7.51), which was used as a positive control. 

**Figure 2:**
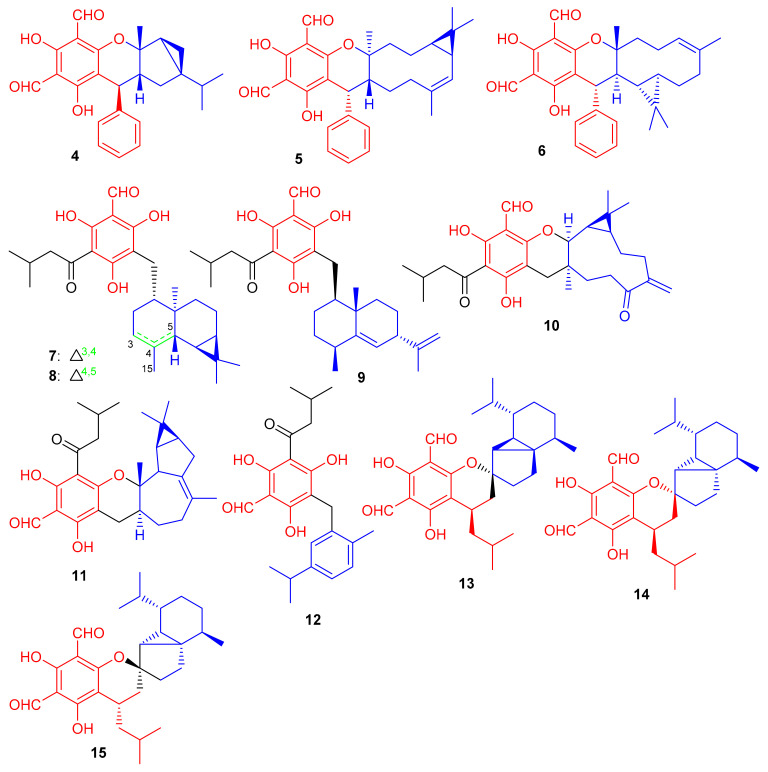
Structures of phloroglucinol-based meroterpenoids **4**–**15**.

Diformylphloroglucinol-based meroterpenoids, viz., guajavadials A-C (**4**–**6**) (Figure 2) were obtained from *P. guajava* and also exhibited cytotoxicity against A-549, HL-60, MCF-7, SMMC-7721, and SW480 cancer cell lines with IC_50_ values between 2.28–3.38 µM (Table 1). Compound **6** displayed the highest potential (IC_50_ = 3.54 µM) against SMMC-7721 cell lines which is higher than the standard cisplatin (IC_50_ = 19.8 µM) [35]. The structures and activity differences revealed that the arrangement of the isoprene units is responsible for the activity potential, and thus the terpenoidal skeleton indeed plays a significant role in the activity level, as can be seen in compounds **5** and **6** [35]. 

Shang et al. [36] isolated a small range of cytotoxic formylphloroglucinol-derived meroterpenoids; the eucalrobusones A–I (**7**–**15**) (Figure 2) from *Eucalyptus robusta*. In the MTT assay, compound **7** moderately inhibited the growth of HepG2 (IC_50_: 18.5 μM) and U2OS (IC_50_: 45.0 μM), while metabolite **10** possesses a weaker potential against HepG2 (26.7 μM) (Table 1). Compound **15** only exhibited moderate inhibition of U2OS cell lines with an IC_50_ value of 42.25 μM. Only metabolite **9** proved to be the most potent anticancer agent against the three target cancer cells with IC_50_: of 7.40 to 8.99. Activity of compound **9** has been reported to be comparable to doxorubicin (IC_50_ = 5.23, 2.66 and 1.14 μM, respectively). A study on the mechanism of action of compound **9** revealed that it significantly inhibited cell division exerting cell proliferation on MCF-7 in a concentration dependent manner. Eucalrobusones A (**7**) and B (**8**) bearing an unusual skeleton having a maaliane sesquiterpene core is linked to a phloroglucinol unit. On the other hand, the chroman ring in eucalrobusone E (**11**) is attached to a bicyclogermacrane unit at the C-3/C-4 position. Meroterpenoid **12** bearing a phloroglucinol unit is linked to an aromatic dehydromenthane monoterpene group. Eucalrobusones G–I (**13**–**15**) are cubebane-based phloroglucinol-based meroterpenoids linked through a 1-oxaspiro[5.5]undecane unit [36].

**Figure 3:**
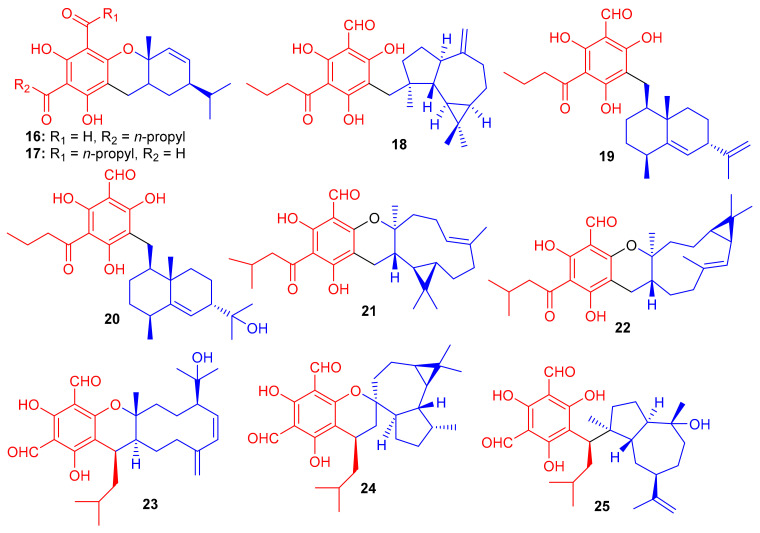
Structures of phloroglucinol-based meroterpenoids **16**–**25**.

Compounds (**16**–**20**) (Figure 3) were obtained from *Eugenia umbelliflora* belonging to the Myrtaceae family and tested for cytotoxic effects towards murine melanoma cells (B16F10 cell) and the leukemic cells (Nalm-6 cells and k562) (Table 1). Interestingly, compound **18** displayed the highest cytotoxicity against K562 cells with an IC_50_ value of 0.38 μM, while compounds **19** and eugenials E (**20**) exhibited inhibitory activity towards K562 cells with IC_50_ values of 1.9 and 4.97 μM, respectively. Compounds **18**–**20** inhibited the growth of B16F10 cells with IC_50_ values of 6.0, 3.2 and 8.8 μM, respectively [37]. Eugenials C–E (**18**–**20**) showed lower inhibitory potential against Nalm-6 cells (Table 1). On the other hand, compound **16** (IC_50_ = 30.5 μM) has been reported to be more potent than **17** (IC_50_ = 42.8 μM) against K562 cells (Table 1). 

Compounds **16** and **17** have fused phloroglucinol-monoterpene systems, while compounds **18**–**20** do not have similar fused systems but only phloroglucinol-sesquiterpene attachments. Therefore, higher activity of compounds **18**–**20** can be attributed to the presence of the additional free phenol groups in their structures. Amongst eugenials C–E (**18**–**20**), the other interesting factor affecting the activity level is their sesquiterpenyl derivatives. Compound **18** (IC_50_ = 0.38 μM), which has a tricyclic aromadendrene core, enhances the activity of this compound by 5-fold and 13-fold respectively, which is encouragingly higher than compound **19** (IC_50_ = 1.9 μM) and compound **20** (IC_50_ = 4.97 μM) [37]. 

*Eucalyptus tereticornis* is reported to produce formyl phloroglucinol meroterpenoids, since Liu et al. [38] isolated five formyl phloroglucinol-derived meroterpenoids viz., eucalteretials A–E (**21**–**25**). Besides spectroscopic analysis, ECD calculations were used to define the chirality of these compounds. At a concentration of 50 μM, compound **25** exhibited comparable topoisomerase I (Top1) inhibitory activity to that of camptothecin, whereas, only compound **23** displayed growth inhibition of HCT116 cell lines with an IC_50_ value of 4.8 μM (Table 1). Among compounds **21**–**25**, both **21** and **22** are rare natural products in which the germacrene core unit and the phloroglucinol core are connected in a different pattern (Figure 3) [38]. 

**Figure 4:**
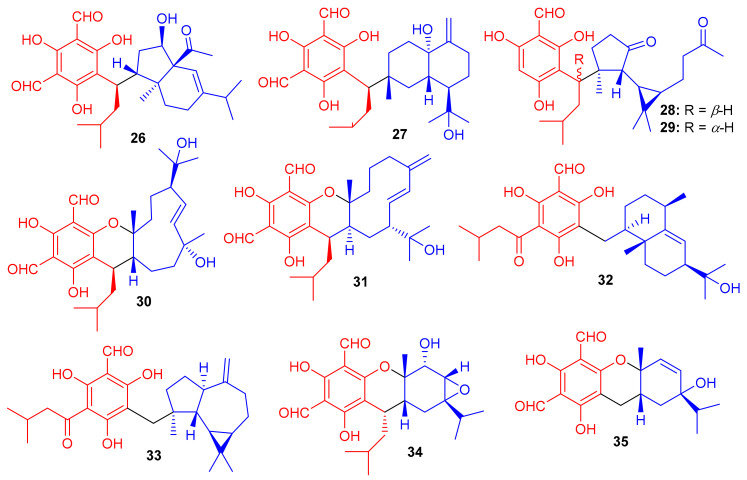
Structures of phloroglucinol-based meroterpenoids **26**–**35**.

*Eucalyptus globulus* fruits are also rich in phloroglucinol-derived meroterpenoids, since several have been isolated previously. Indeed, Qin et al. [39] isolated 10 compounds from this source. The spectroscopic data and ECD calculations lead to their absolute structure determination as eucalypglobulusals A–J (**26**–**35**) (Figure 4) [39]. Eucalypglobulusal A (**26**) has an unusual structure bearing a phloroglucinol core coupled to a rearranged sesquiterpene skeleton. Among these compounds, eucalypglobulusal F (**31**) inhibited the growth of the human acute lymphoblastic cell line (CCRF-CEM) with an IC_50_ value of 3.3 μM (Table 1), which is comparable with the positive control VP-16 (IC_50_ = 1.1 μM). However, the same compound was not active (IC_50_: > 10 μM) towards HCT116, DU145, Huh7 and A549. Moreover, eucalypglobulusal A (**26**) inhibited DNA topoisomerase I (Top1) [39]. 

**Figure 5:**
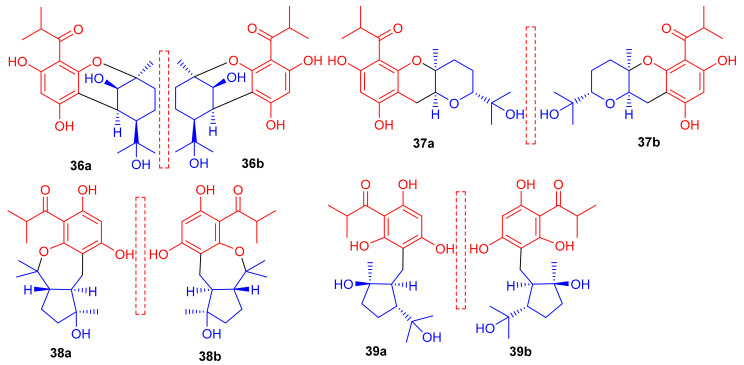
Structures of phloroglucinol-based meroterpenoids **36**–**39**.

Phloroglucinol-based meroterpenoids in the form of enantiomers viz., (±)-japonicols A–D (**36a**,**b**–**39a**,**b**) (Figure 5) were isolated from *Hypericum japonicum*. Moreover only compound **37a** illustrated good KSHV activity (EC_50_: 8.75 μM; SI: 16.0) while the other compounds exhibited EC_50_ values of between 17.6 and 202.9 μM). Biosynthetically, it is suggested that these acylphloroglucinol-derived meroterpenoids are formed by non-enzymatic reactions, since all were isolated from *H. japonicum* in both enantiomeric forms [40]. 

**Figure 6:**
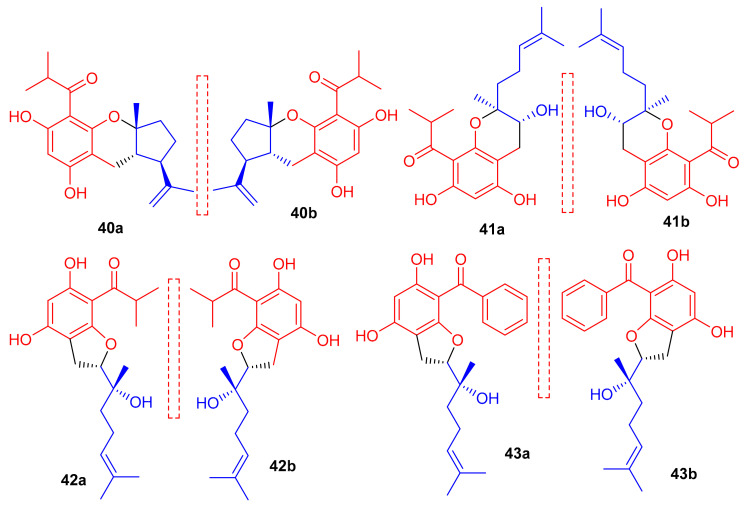
Structures of phloroglucinol-based meroterpenoids **40**–**43**.

New acylphloroglucinol-based meroterpenoid enantiomers viz., (+)-japonicols E–H (**40a**/**b**–**43a**/**b** (Figure 6) have been reported from *H. japonicum* and their structures were established via extensive NMR and ECD techniques. Among these compounds, meroterpenes **40a** and **40b** bore the rare ring unit, cyclopenta[*b*]chromene. Compounds **40a** (IC_50_: 8.30 μM; SI: 23.4) and **43a** (IC_50_: 4.90 μM; SI: 25.7) exhibited inhibitory effects on KSHV (Table 1) [41]. On the other hand, the enantiomers of compounds **40a** and **43a** viz., **40b** (IC_50_: 24.4 μM and **43b** (IC_50_: 29.4 μM) were not especially that active towards KSHV. In addition compound **42b** illustrated inhibitory effects with IC_50_: 6.7 μM and SI: 7.4 while the enantiomer, viz., compound **42a** was not active (IC_50_: 21.3 μM). The authors believe this is illustrative that stereochemistry plays a definite role in enhancing KSHV inhibition. Notably, the activity of **42a** is increased and the selectivity of **43a** is due to the unique phenyl group at C-7′ [41]. 

**Figure 7:**
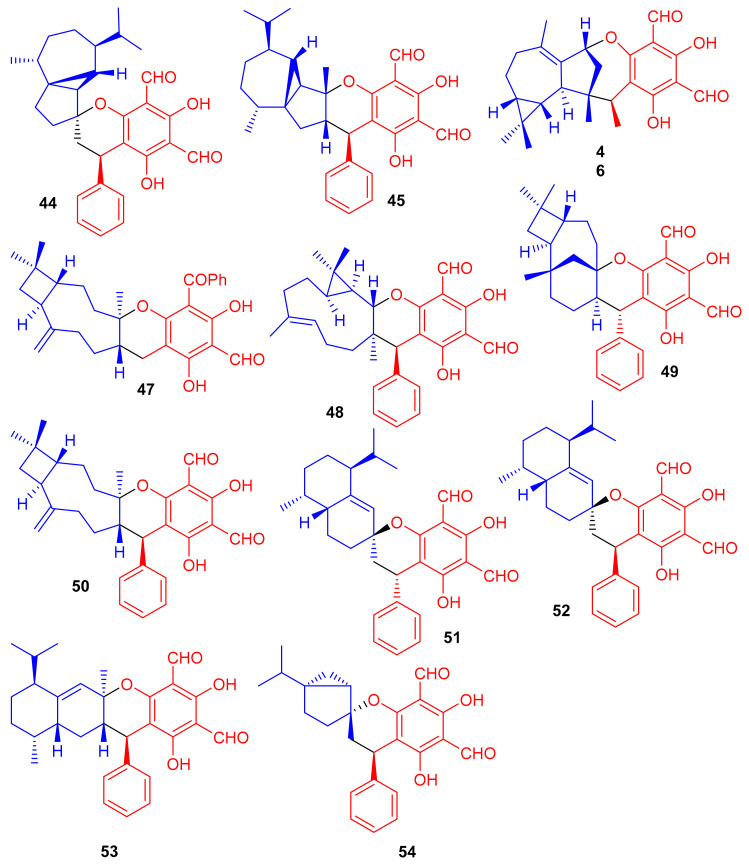
Structures of phloroglucinol-based meroterpenoids **44**–**54**.

Phosphodiesterase-4 (PDE4D-4) inhibitors; psiguajadials A–K (**44**–**54**) (Figure 7) were isolated from *P. guajava*. All these natural products have been reported to be significant inhibitors of PDE4D-4 with IC_50_ values in the range of 1.34–7.26 μM (Table 1) [42]. This activity potential is comparable with the positive control rolipram, a standard PDE4 inhibitor (IC_50_ 0.62 μM). Since a small difference has been reported in the activity level of all these compounds, it may lead to the conclusion that the diformylphloroglucinol moiety is required for PDE4D2 inhibitory activity. The genus *Psidium* produced a diverse range of meroterpenoids bearing phloroglucinol-coupled to sesquiterpenoids or monoterpenes. As illustrative of this variety, consider phloroglucinol-coupled to the cubebane sesquiterpenoid core to produce compounds **44** and **45**, and compound **46** has globulane as the terpene unit while **49** has caryolane and **50** has caryophyllane, whereas compounds **51**–**53** have cadinane as the terpene unit [42]. 

**Figure 8:**
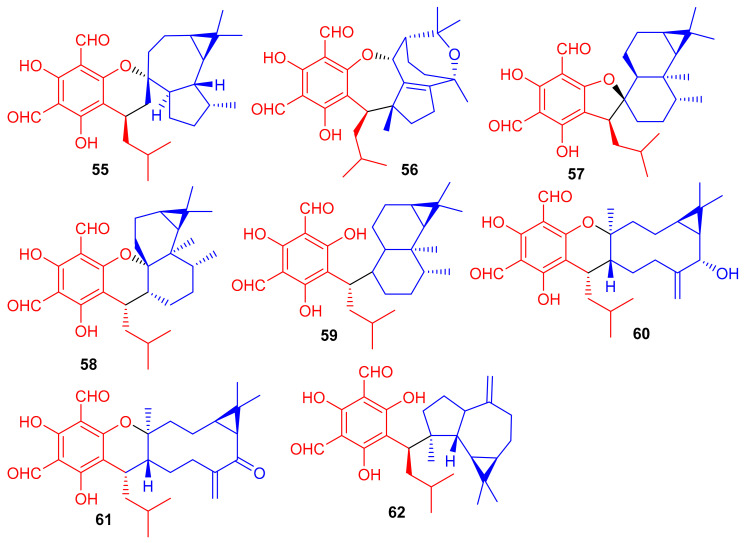
Structures of phloroglucinol-based meroterpenoids **55**–**62**.

Phytomeroterpenoid eucalrobusones J–P (**55**–**61**) and compound **62** (Figure 8) were isolated from *Eucalyptus robusta* and evaluated for their antifungal activity against *C. albicans* and *C. glabrata* [43]. Structural diversity among the meroterpenoids **55**–**62** is generated through the wide range of coupling patterns between the sesquiterpenoid and phloroglucinol units. Meroterpene **55** bearing an unusual carbon skeleton, viz., the 1-oxaspiro[5.6]dodecane unit is formed via the aromadendrane core C-14 rather than C-4. On the other hand, metabolite **56** is a guaiane based meroterpene and interestingly, this compound has two oxo bridges between C-10/C-11 and C-3′/C-6 which generates the most unusual polycyclic ring system [43]. Moreover, meroterpenes **57**–**59** are rare aristolane-based meroterpenoids with only a few examples being reported to date [44]. These metabolites showed different microbial inhibitory potentials (Table 1). Compounds **60** and **62** were moderately active against *C. albicans* with MIC values of 12.54 and 12.50 µg/mL respectively, while other members exhibit MIC values of more than 50 µg/mL. Compounds **55**, **60** and **62** potentially inhibited the growth of *C. glabrata* with MIC values determined as 2.57, 1.95, and 2.49 µg/mL, respectively (Table 1) [43] and these activates are closer to the standard drugs fluconazole (MIC = 0.25 µg/mL) and amphotericin B (MIC = 0.26 µg/mL).

**Figure 9:**
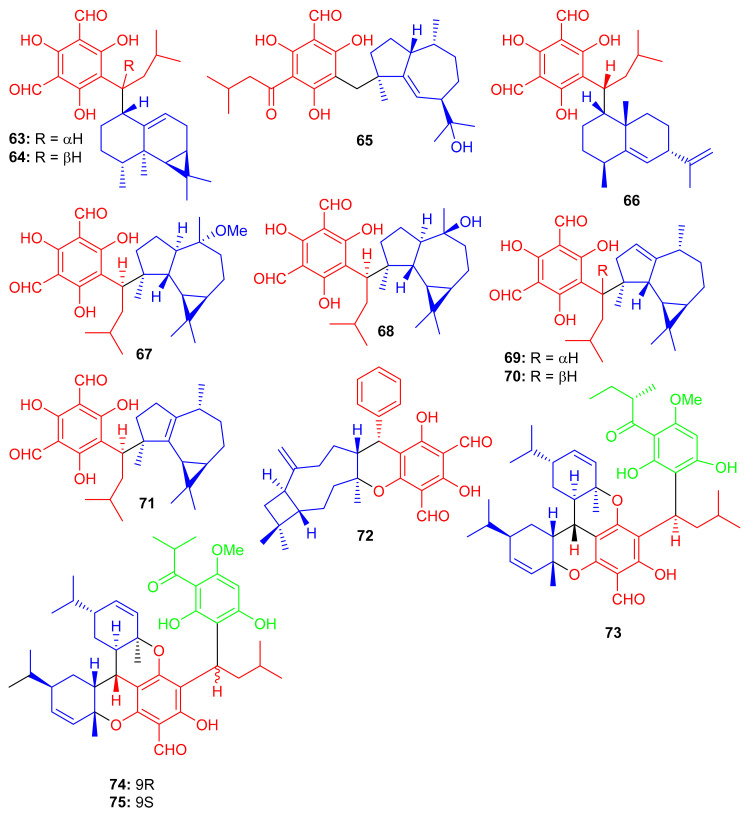
Structures of phloroglucinol-based meroterpenoids **63**–**75**.

*Eucalyptus robusta* produces eucarobustols A–I (**63**–**71**) (Figure 9), which have been identified as PTP1B inhibitors, since all these isolates displayed significant inhibitory potential (IC_50_ = 1.3–5.6 μM, Table 1). In this assay, the standard compound oleanolic acid inhibited the enzyme activity with an IC_50_ value of 2.34 μM [44]. The published results (Table 1) revealed that compounds **63** (IC_50_: 1.3 μM), **67** (IC_50_: 1.8 μM), and **85** (IC_50_: 1.6 μM) were even more potent than oleanolic acid. It is speculated from the cases of the two pairs of epimers (**63**/**64** and **69**/**70**) that the relative configuration of H-9′ can play a central role in the activity and that this provides useful information for further investigations into a structure-activity relationship [44]. Metabolite **65** displays an unusual coupling moiety of acylphloroglucinol and guaiane through the C-4–C-7′ bond. Compound **63** has an acylphloroglucinol coupled sesquiterpene viz., aristolane-type while compounds **67**–**71** have aromadendrane type sesquiterpene linked to acylphloroglucinol units. Guavadial (**72**), isolated from *Psidium guajava* L. has caryophyllene attached to a diformyl phloroglucinol core [45]. Eucalyptusdimers A–C (**73**–**75**) were reported from *Eucalyptus robusta* and were identified via intensive spectroscopic methods. These compounds were shown to bear a fused skeleton between two acylphloroglucinol and two phellandrene cores. Furthermore only compound **73** illustrated anti-AChE effects with an IC_50_: 17.71 μM [46].

**Figure 10:**
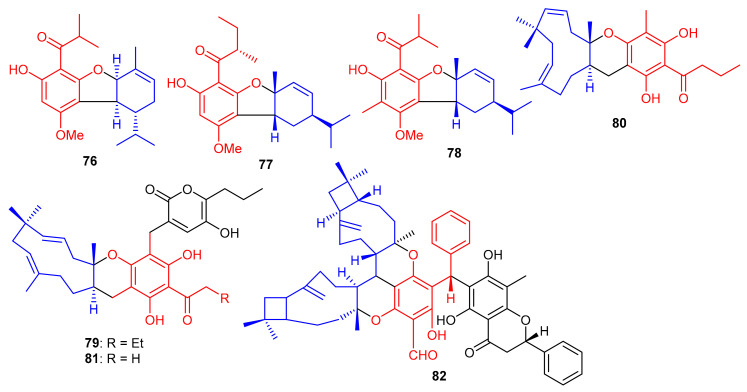
Structures of phloroglucinol-based meroterpenoids **76**–**82**.

**Figure 11:**
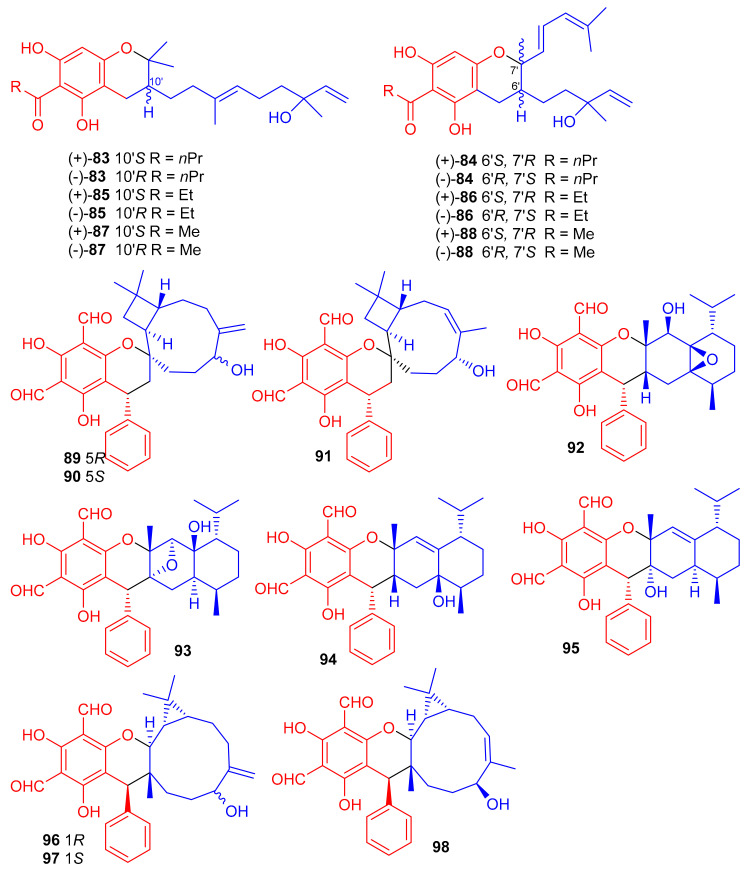
Structures of phloroglucinol-based meroterpenoids **83**–**98**.

Meroterpenoids, callisalignenes A–C (**76**–**78**) (Figure 10) were produced by *Callistemon salignus* and these compounds were not active in antimicrobial screening [47]. Meroterpenoids, drychampones A–C (**79**–**81**), were produced by *Dryopteris championii*. Moreover compounds **80** and **81** featuring an 11/6/6 core coupled with a pyronone moiety [48]. Guajavadimer A (**82**) was produced by *Psidium guajava* and illustrated moderate hepatoprotective effects. Moreover guajavadimer A (**82**) featured unusual dimeric meroterpenoids bearing two caryophyllene skeletons coupled with a benzylphloroglucinol along with flavonone unit [49].

(±)-Dryocrassoids E–J [(±)-**83**–**88**], (Figure 11) were reported from *Dryopteris crassirhizoma* and these metabolites illustrated moderate anti-HSV-1, and anti-RSV effects [50]. Psiguadiols A–J (**89**–**98**) were produce by *Psidium guajava* and evaluated for PTP1B inhibition. Moreover, compounds **83** (IC_50_: 4.7 μM), **95** (IC_50_: 6.2 μM), and **96** (IC_50_: 9.2 μM) demonstrated significant PTP1B inhibition while meroterpenes **90**–**92** illustrated good inhibition with IC_50_ of 11.0, 11.9, and 10.7 μM, respectively. On the other hand the remaining compounds possess PTP1B inhibition with IC_50_ of <23.0 while all compounds were more potent than standard oleanolic acid (IC_50_: 40.7 μM) [51].

**Figure 12:**
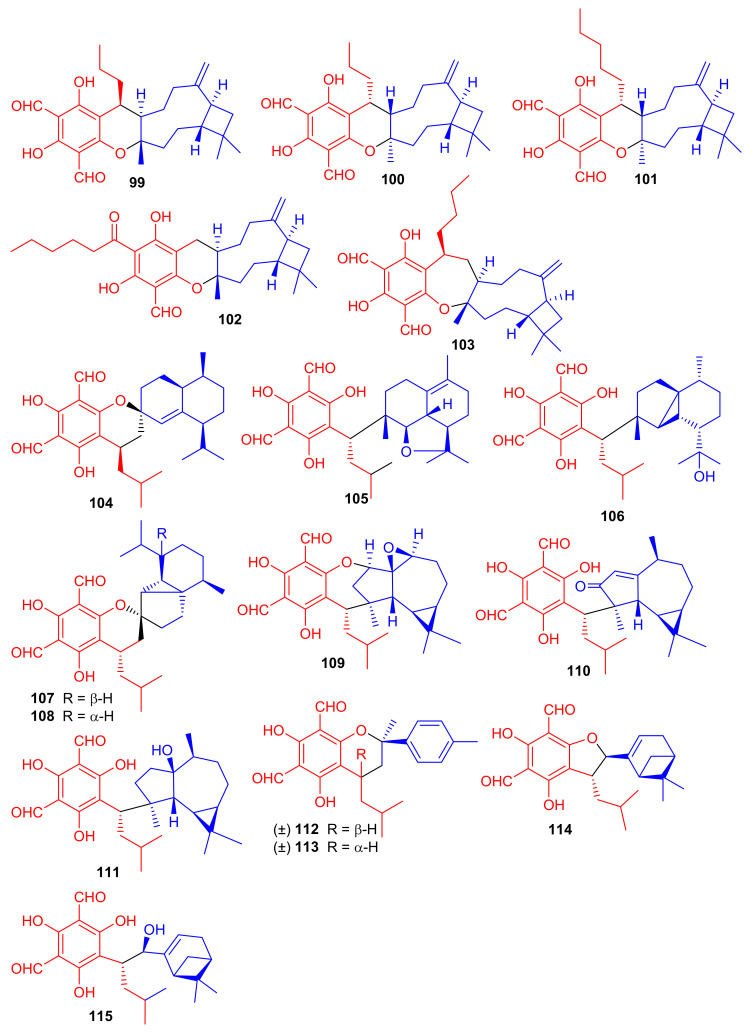
Structures of phloroglucinol-based meroterpenoids **99**–**115**.

Littordials A–E (**99**–**103**), (Figure 12) phloroglucinol-β-caryophyllene coupled meroterpenoids were produce by *Psidium littorale*. Moreover, meroterpenes **100**, **101** and **103** illustrated cytotoxic effects towards B16 and MDA-MB-231 with IC_50_: ranging from 6.6 to 9.2 μM [52]. Meroterpenes, eucalrobusone Q–Z (**104**–**115**) were isolated from *Eucalyptus robusta* and these metabolites have terpene moieties such as cadinane, aromadendrane, cubebane, pinane and aromatic menthane. (+)-Eucalrobusone X (**113**) illustrated the most significant antifungal effects towards *Candida albicans* (MIC_50_: 10.7 μg/mL) while eucalrobusone U (**109**) demonstrated the highest anti-*C. glabrata* effects with MIC_50_: 1.53 μg/mL [53].

**Figure 13:**
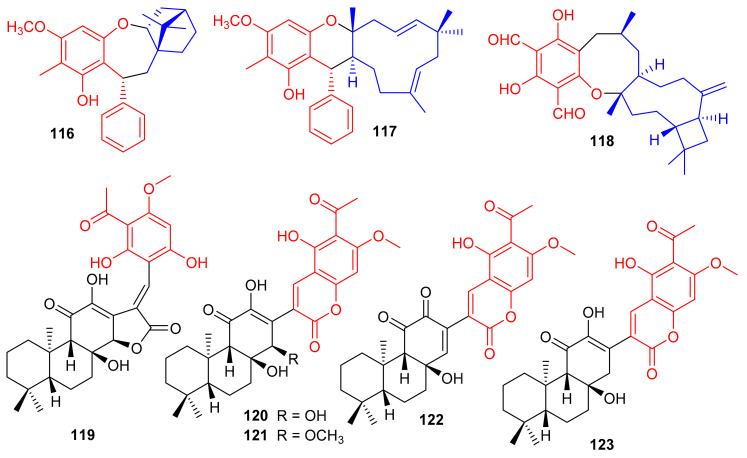
Structures of phloroglucinol-based meroterpenoids **116**–**123**.

Melaleucadines A (**116**) and B (**117**), (Figure 13) phloroglucinol-terpene meroterpenoid metabolites have the phloroglucinol unit coupled with β-pinene to form the humulene-type sesquiterpenes respectively. Moreover, meroterpenes **116** and **117** illustrated a significant neuroprotective effect with cell viability of 53.7% and 58.3%, respectively [54]. Littordial F (**118**) was isolated from *Psidium littorale* which has an unusual 6/8/9/4-tetracyclic core. This compound exhibited moderate cytotoxic effects on B16, MDA-MB-321, and A549 cells with IC_50_ ranging from 21.5 to 41.5 µM [55]. Phloroglucinol based meroterpenes named fischernolides A–D (**119**–**122**), having a diterpene core (Figure 13), were produced by *Euphorbia fischeriana*. Meroterpenes 120 and 122 illustrated significant cytotoxic effects towards HT-29, Bel-7402, MCF-7, A549, and HeLa with IC_50_ values from 2.0 to 8.6 μM [56]. Euphoractone (**123**) was isolated from *Euphorbia fischeriana* and displayed cytotoxic effects towards H23 and H460 cells with the IC_50_: 21.0 and 20.9 mmol/L respectively [57].

**Table 1:** Phloroglucinol-Derived Metroterpenoids and their biological effects.

Compounds	Source	Activities	Ref.
Psiguajavadial A (**1**)	*Psidium guajava*	**Cytotoxic effects**: HCT116 = IC_50_ 7.60 µM; CCRF-CEM = IC_50_ 25.2 µM; DU145 = IC_50_ 20.2 µM; Huh7 = IC_50_ 48.8 µM; A549 = IC_50_ 2.99 µM	[33,34]
Psiguajavadial B (**2**)	*Psidium guajava*	**Cytotoxic effects**: HCT116 = IC_50_ 21.6 µM; CCRF-CEM = IC_50_ 9.63 µM; DU145 = IC_50_ 26.3 µM; Huh7 = IC_50_ 13.7 µM; A549 = IC_50_ 0.90 µM	[33,34]
Guadial A (**3**)	*Psidium guajava*	**Cytotoxic effects**: HCT116 = IC_50_ 5.74 µM; CCRF-CEM = IC_50_ 2.95 µM; DU145 = IC_50_ 5.35 µM; Huh7 = IC_50_ 28.0 µM; A549 = IC_50_ 9.62 µM; **Enzyme Inhibition**: PDE4D-4 = IC_50_ 2.70 μM	[33,34]
Guajavadial A (**4**)	*Psidium guajava*	**Cytotoxic effects**: HL-60 = IC_50_ 4.73 µM; A-549 = IC_50_ 5.62 µM; SMMC-7721 = IC_50_ 4.37 µM; MCF-7 = IC_50_ 22.28 µM; SW480 = IC_50_ 14.55 µM; **Enzyme Inhibition**: PDE4D-4 = IC_50_ 2.01 μM	[35]
Guajavadial B (**5**)	*Psidium guajava*	**Cytotoxic effects**: HL-60 = IC_50_ 6.49 µM; A-549 = IC_50_ 5.78 µM; SMMC-7721 = IC_50_ 5.05 µM; MCF-7 = IC_50_ 18.02 µM; SW480 = IC_50_ 13.07 µM	[35]
Guajavadial C (**6**)	*Psidium guajava*	**Cytotoxic effects**: HL-60 = IC_50_ 3.38 µM; A-549 = IC_50_ 5.66 µM; SMMC-7721 = IC_50_ 3.54 µM; MCF-7 = IC_50_ 14.54 µM; SW480 = IC_50_ 18.97 µM	[35]
Eucalrobusone A (**7**)	*Eucalyptus robusta*	**Cytotoxic effects**: HepG2 = IC_50_ 18.52 µM; U2OS = IC_50_ 45.00 μM	[36]
Eucalrobusone C (**9**)	*Eucalyptus robusta*	**Cytotoxic effects**: HepG2 = IC_50_ 7.40 µM; U2OS = IC_50_ 8.99 μM; MCF-7 = IC_50_ 8.50 μM	[36]
Eucalrobusone D (**10**)	*Eucalyptus robusta*	**Cytotoxic effects**: HepG2 = IC_50_ 26.78 μM	[36]
Eucalrobusone H (**14**)	*Eucalyptus robusta*	**Cytotoxic effects**: U2OS = IC_50_ 42.25 μM	[36]
Eugenial B (**17**)	*Eugenia umbelliflora*	**Cytotoxic effects**: K562 = IC_50_ 42.8 μM; Nalm-6 = IC_50_ 70.5 μM; B16F10 = IC_50_ 12.0 μM	[37]
Eugenial C (**18**)	*Eugenia umbelliflora*	**Cytotoxic effects**: K562 = IC_50_ 0.38 μM; Nalm-6 = IC_50_ 10.5 μM; B16F10 = IC_50_ 6.0 μM	[37]
Eugenial D (**19**)	*Eugenia umbelliflora*	**Cytotoxic effects**: K562 = IC_50_ 1.90 μM; Nalm-6 = IC_50_ 7.75 μM; B16F10 = IC_50_ 3.20 μM	[37]
Eugenial E (**20**)	*Eugenia umbelliflora*	**Cytotoxic effects**: K562 = IC_50_ 4.97 μM; Nalm-6 = IC_50_ 29.1 μM; B16F10 = IC_50_ 8.80 μM	[37]
Eucalteretials C (**23**)	*Eugenia tereticornis*	**Cytotoxic effects**: HCT116 = IC_50_ 4.8 μM	[37]
Eucalypglobulusal F (**31**)	*Eugenia globulus*	**Cytotoxic effects**: CCRF-CEM = IC_50_ 3.3 μM	[37]
(+)-Japonicol B (**37a**)	*Hypericum japonicum*	**Antiviral effects**: KSHV = EC_50_ 8.75 μM	[40]
(+)-Japonicol E (**40a**)	*Hypericum japonicum*	**Antiviral effects:** KSHV = IC_50_: 8.30 μM	[41]
(−)-Japonicol E (**40b**)	*Hypericum japonicum*	**Antiviral effects**: KSHV = IC_50_: 24.4 μM	[41]
(+)-Japonicol G (**42a**)	*Hypericum japonicum*	**Antiviral effects**: KSHV = IC_50_: 21.3 μM	[41]
(−)-Japonicol G (**42b**)	*Hypericum japonicum*	**Antiviral effects**: KSHV = IC_50_: 6.7 μM	[41]
(+)-Japonicol H (**43a**)	*Hypericum japonicum*	**Antiviral effects**: KSHV = IC_50_: 4.90 μM	[41]
(−)-Japonicol H (**43b**)	*Hypericum japonicum*	**Antiviral effects**: KSHV = IC_50_: 29.4 μM	[41]
Psiguajadial A (**44**)	*Psidium guajava*	**Enzyme Inhibition**: PDE4D-4 = IC_50_ 3.11 μM	[42]
Psiguajadial B (**45**)	*Psidium guajava*	**Enzyme Inhibition**: PDE4D-4 = IC_50_ 5.03 μM	[42]
Psiguajadial C (**46**)	*Psidium guajava*	**Enzyme Inhibition**: PDE4D-4 = IC_50_ 4.50 μM	[42]
Psiguajadial D (**47**)	*Psidium guajava*	**Enzyme Inhibition**: PDE4D-4 = IC_50_ 4.14 μM	[42]
Psiguajadial E (**48**)	*Psidium guajava*	**Enzyme Inhibition**: PDE4D-4 = IC_50_ 3.25 μM	[42]
Psiguajadial F (**49**)	*Psidium guajava*	**Enzyme Inhibition**: PDE4D-4 = IC_50_ 2.63 μM	[42]
Psiguajadial G (**50**)	*Psidium guajava*	**Enzyme Inhibition**: PDE4D-4 = IC_50_ 1.34 μM	[42]
Psiguajadial H (**51**)	*Psidium guajava*	**Enzyme Inhibition**: PDE4D-4 = IC_50_ 1.81 μM	[42]
Psiguajadial I (**52**)	*Psidium guajava*	**Enzyme Inhibition**: PDE4D-4 = IC_50_ 2.51 μM	[42]
Psiguajadial J (**53**)	*Psidium guajava*	**Enzyme Inhibition**: PDE4D-4 = IC_50_ 2.53 μM	[42]
Psiguajadial K (**54**)	*Psidium guajava*	**Enzyme Inhibition**: PDE4D-4 = IC_50_ 3.68 μM	[42]
Psiguadial A (**55**)	*Psidium guajava*	**Enzyme Inhibition**: PDE4D-4 = IC_50_ 7.26 μM	[42]
Guapsidial A (**56**)	*Psidium guajava*	**Enzyme Inhibition**: PDE4D-4 = IC_50_ 5.61 μM	[42]
Psiguajadial L (**57**)	*Psidium guajava*	**Enzyme Inhibition**: PDE4D-4 = IC_50_ 1.37 μM	[42]
Eucarobustol A (**63**)	*Eucalyptus robusta*	**Enzyme Inhibition**: PTP1B = IC_50_ 1.3 μM	[44]
Eucarobustol B (**64**)	*Eucalyptus robusta*	**Enzyme Inhibition**: PTP1B = IC_50_ 4.3 μM	[44]
Eucarobustol C (**65**)	*Eucalyptus robusta*	**Enzyme Inhibition**: PTP1B = IC_50_ 4.3 μM	[44]
Eucarobustol D (**66**)	*Eucalyptus robusta*	**Enzyme Inhibition**: PTP1B = IC_50_ 2.9 μM	[44]
Eucarobustol E (**67**)	*Eucalyptus robusta*	**Enzyme Inhibition**: PTP1B = IC_50_ 4.1 μM	[44]
Eucarobustol F (**68**)	*Eucalyptus robusta*	**Enzyme Inhibition**: PTP1B = IC_50_ 5.6 μM	[44]
Eucarobustol G (**69**)	*Eucalyptus robusta*	**Enzyme Inhibition**: PTP1B = IC_50_ 1.8 μM	[44]
Eucarobustol H (**70**)	*Eucalyptus robusta*	**Enzyme Inhibition**: PTP1B = IC_50_ 3.0 μM	[44]
Eucarobustol I (**71**)	*Eucalyptus robusta*	**Enzyme Inhibition**: PTP1B = IC_50_ 1.6 μM	[44]
Eucalyptusdimers A (**73**)	*Eucalyptus robusta*	**Enzyme Inhibition**: AChE = IC_50_ 17.71 μM	[44]

## 4. Syncarpic Acid/β-Triketones-Based Meroterpenes

**Figure 14:**
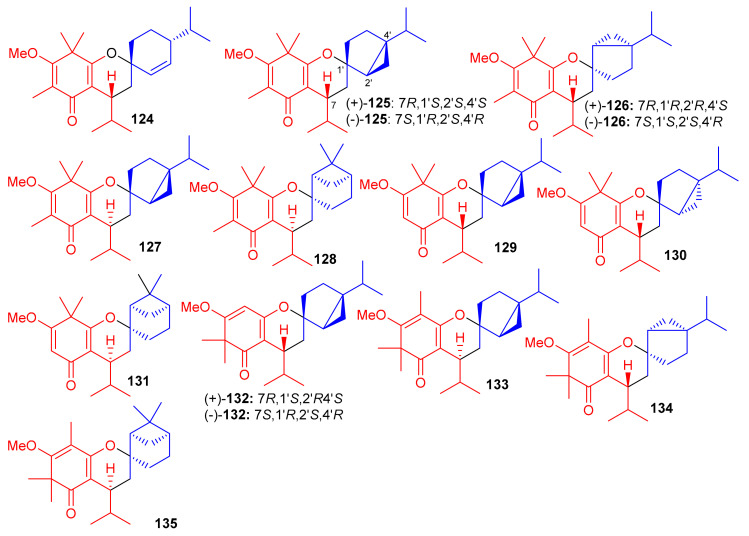
Structures of syncarpic acid/β-triketones-based meroterpenes **124**–**135**.

*Baeckea frutescens* has been reported to produce a small library of polymethylated phloroglucinol meroterpenoids, viz., baeckfrutones A–L (**124**–**135**) (Figure 14). The structures of these metabolites were also elucidated via spectroscopic methods, X-ray diffraction techniques, and ECD calculations [58]. Meroterpenoids **124** and **125**–**127** are novel hybrid compounds of the enone-type phloroglucinols linked with phellandrene and sabinene, respectively. Biogenetically, meroterpenoids **124**–**135** involve regio- and stereoselective [4 + 2] cycloaddition reactions between demethylated tasmanone or tasmanone and monoterpenoids, viz., sabinene, β-phellandrene, and β-piene. Enone-type phloroglucinol (-)-**125** (IC_50_: 1.33 μM) and **133** (IC_50_: 4.04 μM) showed potent inhibition of DU145 cell lines, which is more potent than VP-16, the standard inhibitor (IC_50_ = 5.22 μM). On the other hand, compounds **129** and **134** displayed moderate growth inhibition of A549 (IC_50_: 15.6 μM) and HCT116 (IC_50_: 12.8 μM) cell lines (Table 2). Moreover compounds **129** (74.4%), **130** (75.3%), (+)-**132** (55.1%), and **131** (75%) illustrated significant anti-inflammatory effects. Additionally, compound **133** (IC_50_: 43.0 μM) displayed good effects towards AChE. The remainder of the metabolites were either weakly active or inactive [58].

**Figure 15:**
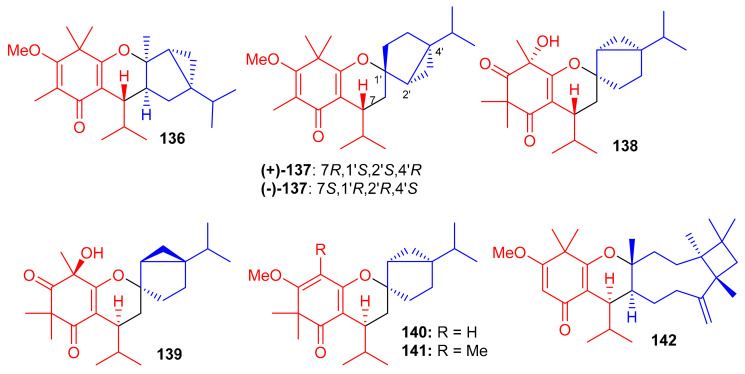
Structures of syncarpic acid/β-triketones-based meroterpenes **136**–**142**.

Further phloroglucinol-derived phytomeroterpenoids, named baeckfrutones M–S (**136**–**142**) (Figure 15), were isolated from *B. frutescens*. Biogenetically, meroterpenoids **136**–**142** also involve a [4 + 2] cycloaddition condensation between demethylated tasmanone or tasmanone and monoterpenoids viz., sabinene, thujene, and caryophyllene [59]. Compounds **136**–**142** were tested for anti-inflammatory activity, in which it was found that only (+)-**136** (IC_50_: 20.8 μM) and **142** (IC_50_: 36.2 μM) displayed potent effects and their activities were more potent than the positive control L-*N*G-monomethyl arginine (L-NMMA, IC_50_ = 54.0 μM). Moreover, none of these compounds were active towards HL-60, A-549, MCF-7, SW480, and SMMC-7721 cancer cells [59].

**Figure 16:**
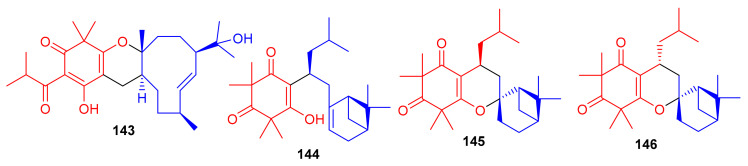
Structures of syncarpic acid/β-triketones-based meroterpenes **143**–**146**.

Hyperjaponol H (**143**) (Figure 16), obtained from *Hypericum japonicum,* was identified with the help of spectroscopic analyses and a comparison of the Cotton effects of an ECD spectrum. Hyperjaponol H (**143**) is a hybrid of tasmanone and the monoterpene germacrane. An assay on lytic DNA replication of EBV in B95-8 cells indicated that this compound displayed moderate inhibitory effects with an EC_50_ value of 25.00 μM [60] (Table 2). Spectroscopic identification of the secondary metabolites of *Rhodomyrtus tomentosa* revealed that tomentosenol A (**144**), 4*S*-focifolidione (**145**) and 4*R*-focifolidione (**146**) contain a unique free syncarpic acid-derived meroterpenoid skeleton. Compound **144** was also confirmed through biomimetic synthesis and was shown to potentially inhibit the growth of *S. aureus* with an MIC value of 4.74 μM, which has been reported to be comparable with the standard drug vancomycin (MIC = 1.23 μM). Since the other compounds **113** and **146** have been reported as being inactive, it seems that the pyran ring is responsible for reducing the antibacterial activity. In addition, tomentosenol A (**144**) moderately inhibited the growth of MCF-7 (IC_50_ = 8.66 μM), NCI-H460 (IC_50_ = 8.62 μM), SF-268 (IC_50_ = 10.01 μM) and HepG-2 (IC_50_ = 9.44 μM) (Table 2) [61].

**Figure 17:**
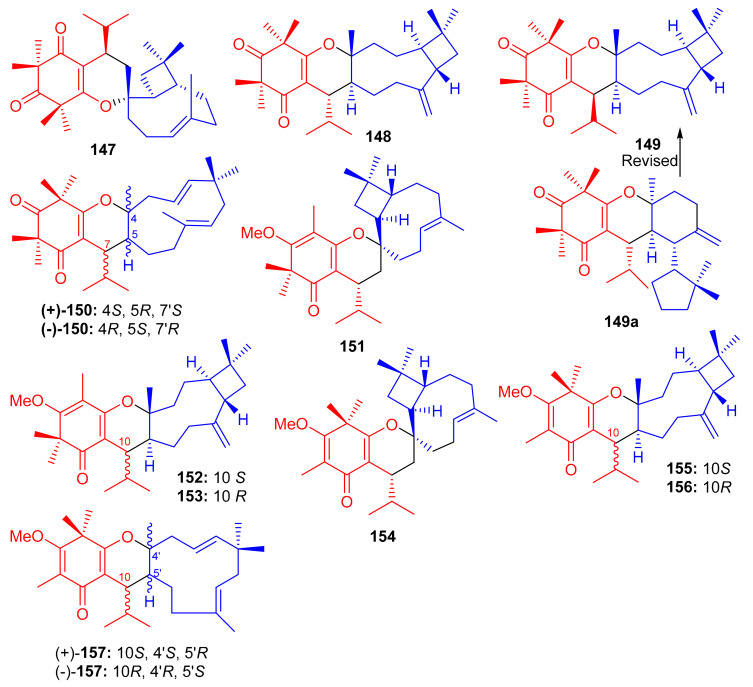
Structures of syncarpic acid/β-triketones-based meroterpenes **147**–**157**.

Liu et al. [62] isolated syncarpic acid-derived meroterpenoids from *Myrtus communis*. Spectroscopic analysis revealed that myrtucommulones (**147**–**149**) (Figure 17) and (±)-**150** having a different skeleton to compound **147** affords a unique octahydrospiro{bicyclo[7.2.0]undecane-2,2′-chromene} tetracyclic ring system. Compounds **147**–**149** bear a syncarpic acid coupled with the sesquiterpene viz., caryophyllene while compound **150** has humulene as the sesquiterpene core [54]. In an MTT assay, compound **147** inhibited the growth of HepG2 (IC_50_: 4.3 μM) and MDA-MB-231 cells (IC_50_: 19.9 μM), whereas metabolite **150** demonstrated IC_50_ values of 40.7 and 40.0 μM, respectively (Table 2). Compounds **149** and **150** were inactive under these conditions [28]. In 2012, Cottiglia et al. [63] reported myrtucommulone K (**149a**) from *M. communis* and based on NMR data, the authors confirmed that the structure of **149** is identical to myrtucommulone K (**149a**).

Frutescone A–G [(**151**–**156**), (+)-**157** and (−)-**157**] were obtained from *Baeckea frutescens* L. and were shown to possess chemical structures similar to compounds **147**–**149**. Compounds **151**–**156** bear a triketone coupled with the sesquiterpene, viz., caryophyllene while compound **150** has humulene as the sesquiterpene core (Figure 17) [64]. Compounds **151** and **154** displayed anticancer activity against Caco-2 with IC_50_ values of 8.08 µM and 10.20 µM, respectively, whereas, compound **155** inhibited the growth of Caco-2 and A549 cell lines with IC_50_ values of 7.96 µM. The other metabolites were only weakly active (Table 2), but all were inactive against HepG2 cells [64].

**Figure 18:**
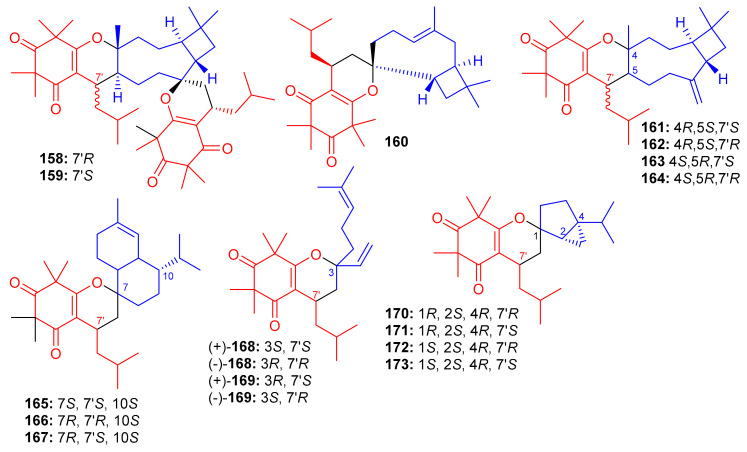
Structures of syncarpic acid/β-triketones-based meroterpenes **158**–**173**.

Triketone-caryophyllene-based meroterpenoids isolated from *Rhodomyrtus tomentosa* were identified as rhodomyrtials A and B (**158** and **159**), rhodomentone A (**160**) and tomentodiones A-D (**161**–**164**) (Figure 18) and all were evaluated for their inhibitory potential on tumor metastasis. Compound **161** has a unique 1-oxaspiro[5,8]tridecane core bearing two units of triketone. Biological evaluation demonstrated that only compound **164** displayed significant metastatic effects towards DLD-1 cells. Since no study has been carried out on the mode of action, it should be mentioned that the stereochemistry at C-7 could play an important role in the activity [65].

Zhang et al. [66] separated the tomentodiones E–M (**165**–**173**) (Figure 18) from an extract of *Rhodomyrtus tomentosa*. It was hypothesized that compounds **165**–**173** could form via a Diels–Alder reaction between triketone and three appropriate terpenes viz., β-calacorene, (+)-sabinene, and myrcene [66]. Since compounds **165**, (±)-**168**, (±)-**169** and **170**–**173** were non-cytotoxic towards doxorubicin-resistant human breast carcinoma cells (MCF-7/DOX), and compound (+)-**168** exhibited a significant potentiation effect by 16.5 reversal fold for MCF-7/DOX, activity was also observed for (+)-**169** (10.1 fold), (−)-**169** (7.4 fold), **173** (5.6 fold), (−)-**168** (4.7 fold), and **169** (4.5 fold). Comparison of the activity and structural features of these compounds, suggests that the stereochemistry at C-7′ might be significant in determining and enhancing biological effects [66].

**Figure 19:**
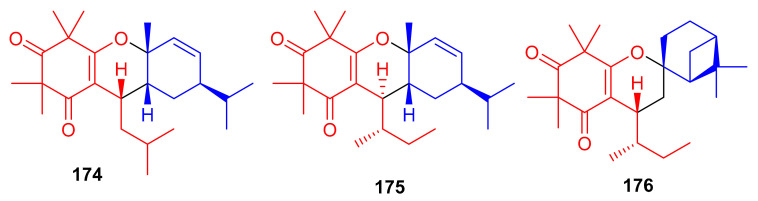
Structures of syncarpic acid/β-triketones-based meroterpenes **174**–**176**.

Callisalignenes G–I (**174**–**176**) (Figure 19), isolated from the medicinal plant *Callistemon salignus*, illustrate β-triketone and monoterpene moieties in their structures. Spectroscopic analysis and CD calculations revealed that compounds **175** and **176** also possess a *sec*-butyl moiety at C-7, which is not so common in natural products. The three metabolites inhibited the growth of HCT116 cancer cell lines with IC_50_ values of 8.51, 9.12 and 16.33 μM, respectively (Table 2). This activity has been reported to be even better than the positive control (VP-16, 20.26 µM). Compounds **174** and **176** also exhibited cytotoxicity against A549 cell lines with IC_50_ values of 12.85 and 10.03 μM, respectively, which is also better than VP-16 (IC_50_ = 25.79 ± 6.2 µM), respectively [67].

**Figure 20:**
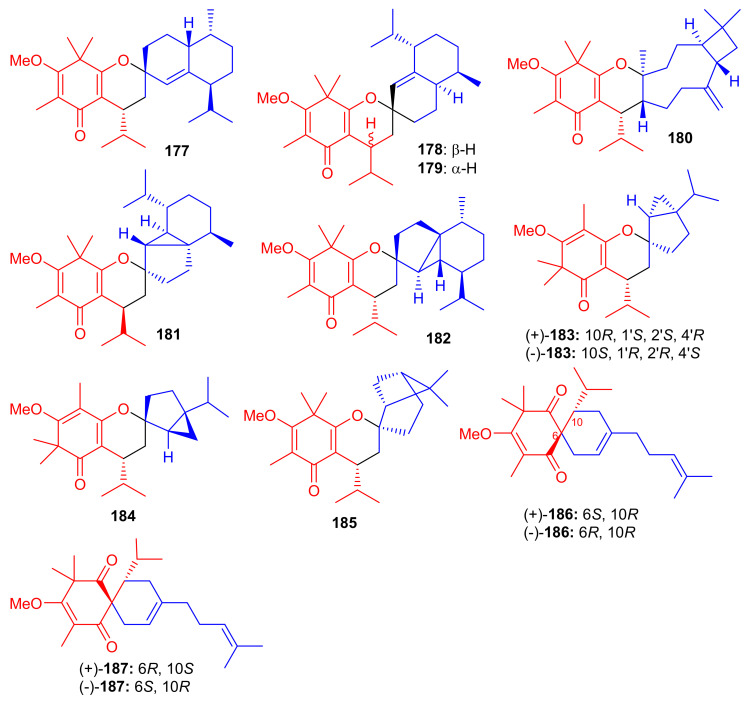
Structures of syncarpic acid/β-triketones-based meroterpenes **177**–**187**.

The small library of compounds **177**–**187** (Figure 20) were separated from *Baeckea frutescens* and identified through spectroscopic analyses, ECD calculations and X-ray crystallography. Metabolites **177**–**187** could form between the common triketone (tasmanone) sesquiterpenes/monoterpenes viz., bicyclosequiphellandrene (compounds **145**–**147**), β-caryophyllene (compound **148**), β-cubebene (compounds **181** and **182**), (−)-sabinene (compounds **183** and **184**), β-pinene (compound **185**), and myrcene (compounds **186** and **187**) [68].

Among other antioxidant meroterpenoids, frutescones H–R (**177**–**182**) only moderately inhibited the NO production in LPS-induced RAW 264.7 cells with IC_50_ values in the range of 15.17–50.0 μM (Table 2), whereas, compounds **183**–**187** exhibited significant IC_50_ values (0.36–6.50 μM) [68]. These compounds are more potent inhibitors of NO production than the standard *N*-monomethyl-L-arginine (L-NMMA, IC_50_ = 30.92 μM). Since compound **184** showed the highest anti-inflammatory potential (IC_50_ = 0.36 μM), it was further evaluated against LPS-induced upregulation of TNF-α and IL-6. A review of the activity level and structural features of compounds **177**–**187** revealed that the overall structure of ring A plays a more important role in the potential of these compounds, especially the position of the double bonds and keto groups [68].

**Figure 21:**
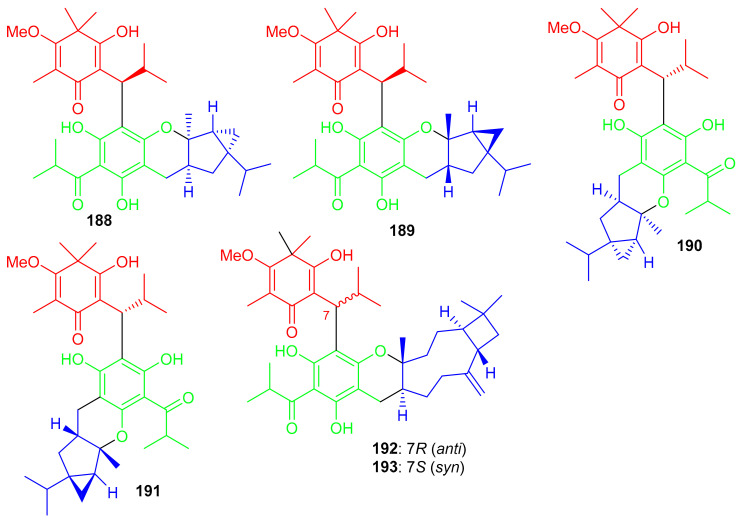
Structures of syncarpic acid/β-triketones-based meroterpenes **188**–**193**.

Baefrutones A–F (**188**–**193**) (Figure 21) bearing a rather rare triketone-phloroglucinol unit coupled to sesquiterpene/monoterpene skeletons were isolated from *Baeckea frutescens*. Interestingly, in compounds **188**–**191**, the triketone-phloroglucinol core was attached to α-thujene while meroterpenoids **192** and **193** have a β-caryophyllene framework instead of the α-thujene. Moreover compounds **188**–**191** displayed anti-inflammatory effects towards NO production with IC_50_ = 9.1 to 18.0 μM (Table 2), while meroterpenoids **192** and **193** were not active. Notably compounds **188**–**191** were more potent than the positive control L-NMMA (IC_50_ = 30.9 μM) [69].

**Figure 22:**
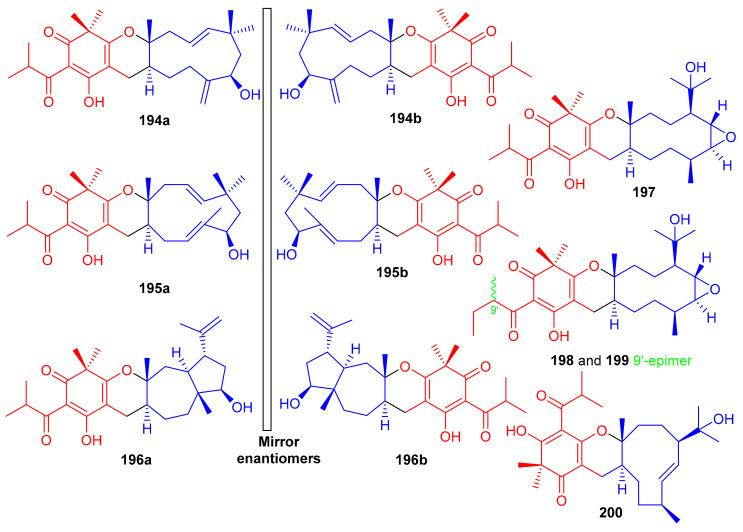
Structures of syncarpic acid/β-triketones-based meroterpenes **194**–**200**.

Filicinic acid-based meroterpenoids, the hyperjaponols A–G (**194**–**200**) (Figure 22) were isolated from *Hypericum japonicum* and comprise 6/6/7/5, 6/6/11, or 6/6/10 sized ring frameworks. Moreover compounds **194**–**196** were reported as enantiomeric pairs and these compounds displayed weak to moderate anti-EBV effects while compound **200** was not active. Enantiomer **194a** displayed an EC_50_ value of 10.33 μM, while its enantiomer **195b** was weakly active (Table 2). In a similar enantiomeric differentiation, metabolite **195a** (EC_50_ = 0.57 μM) was more active than **163b** (EC_50_ = 6.60 μM) (Table 2). Among other isolates, compound **197** exhibited the lowest EC_50_ value of 0.49 μM, which is 5-fold lower than the standard drug ganciclovir (EC_50_ 2.86 μM) [70].

**Figure 23:**
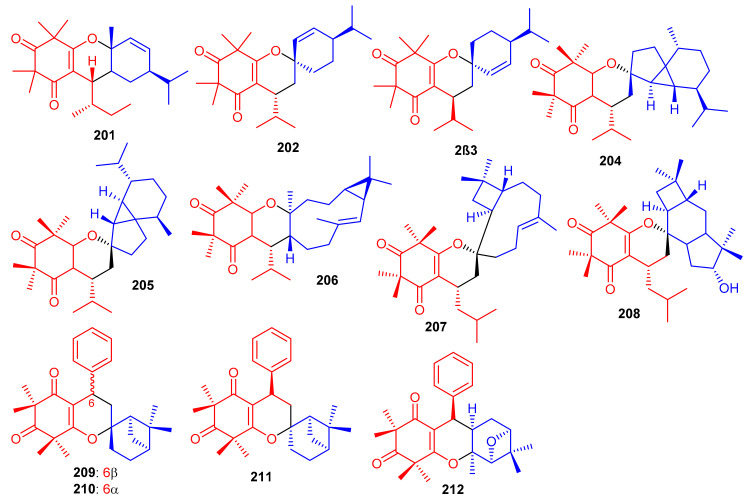
Structures of syncarpic acid/β-triketones-based meroterpenes **201**–**212**.

Meroterpenoids, callisalignenes D–F (**201**–**203**) (Figure 23) were produced by *Callistemon salignus* [47] and based on MIC values [71] these compounds were not active in antimicrobial screening. In another report, meroterpenoids, myrtucomvalones A–C (**204**–**206**) were reported from *Myrtus communis* and compound **206** illustrated moderate antiviral effects towards the respiratory syncytial virus (RSV) with IC_50_: 15.8 µM [72]. Liu et al. [73] reported two meroterpenoids, rhodomentones A (**207**) and B (**208**) were produced by *Rhodomyrtus tomentosa* and featured an uncommon caryophyllene-conjugated oxa-spiro[5.8]tetradecadiene core. In addition, Senadeera et al. [74] reported intermediones A–D (**209**–**212**) from the tree *Corymbia intermedia*. Compounds **209**, **210**, and **212** possessed moderate antiplasmodial effects towards *Plasmodium falciparum* with IC_50_: 12.5, 9.9 to 20.8 μM, respectively.

**Figure 24:**
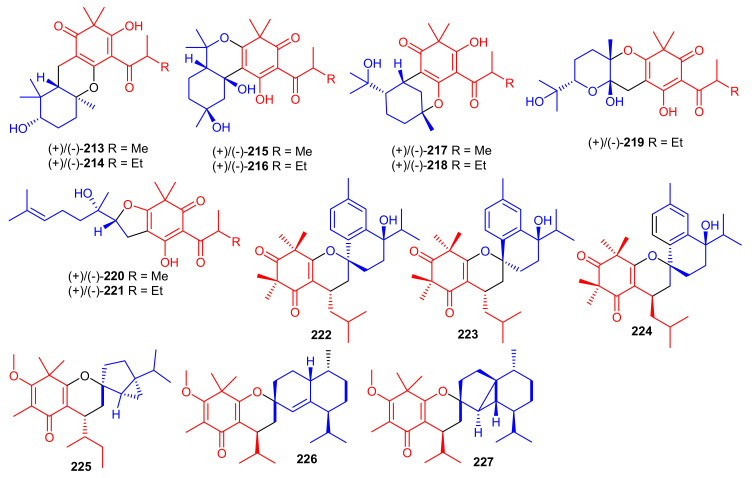
Structures of syncarpic acid/β-triketones-based meroterpenes **213**–**227**.

Elodeoidols A–I (**213**–**221**) (Figure 24) were isolated from *Hypericum elodeoides*. Compounds **217**, **220** and **221** illustrated moderate antibacterial effects towards *Streptococcus mutans*, *Fusobacterium nucleatum*, and *Streptococcus sanguis.* In addition, compounds **215**, **219** and **220** demonstrated potent NO inhibitory effects towards LPS induced RAW264.7 cells with IC_50_ ranging from 10 to 34 μM [75]. Moreover, rhotomentodiones C–E, (**222**–**224**) (Figure 24) were produced by *Rhodomyrtus tomentosa.* Rhotomentodione D (**223**) demonstrated antibacterial effects towards *Propionibacterium acnes* (MIC: 12.5 μg/mL) and AChE inhibitory effects with an IC_50_: 22.9 μM [76]. Frutescones S–U (**225**–**227**) were isolated from *Baeckea frutescens* and meroterpene **225** demonstrated potent anti-inflammatory effects with an IC_50_: 0.81 μmol/L [77].

**Figure 25:**
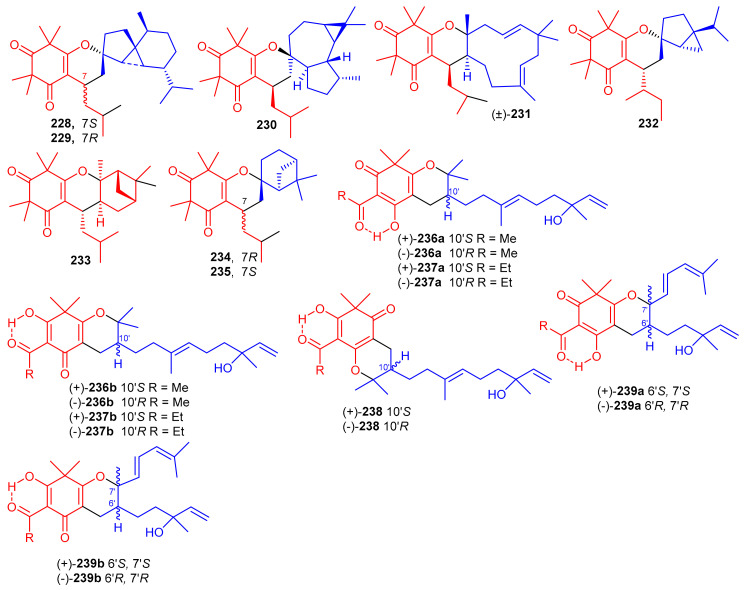
Structures of syncarpic acid/β-triketones-based meroterpenes **228**–**239**.

*β*-Triketone-based meroterpenes rtomentones A–H (**228**–**235**) (Figure 25) were isolated from *Rhodomyrtus tomentosa* and all compounds were not active towards A549, MDA-MB-231, and DLD-1 cancer cells [78]. (±)-Dryocrassoids A–D (**236**–**239**) were reported from *Dryopteris crassirhizoma*. Moreover these compounds exhibited moderate anti-HSV-1 activity with IC_50_: ranging from 23.4 to 95.0 µM. In addition, compounds **236**–**239** also possessed anti-RSV effects with IC_50_: ranging from 11.4 to 50.2 µM. [50].

**Table 2:** Syncarpic acid/β-triketones-based meroterpenes.

Compounds	Source	Anticancer	Ref.
(−)-Baeckfrutone B (**125**)	*Baeckea frutescens*	**Cytotoxic effects**: DU145 = IC_50_ 79.45 μM	[58]
(+)-Baeckfrutone C (**126**)	*Baeckea frutescens*	**Cytotoxic effects**: HCT116 = IC_50_ 62.64 μM; Hela = IC_50_ 85.79 μM; DU145 = IC_50_ 17.65 μM; A549 = IC_50_ 86.68 μM	[58]
(−)-Baeckfrutone C (**126**)	*Baeckea frutescens*	**Cytotoxic effects**: HCT116 = IC_50_ 49.09 μM; Hela = IC_50_ 91.22 μM; DU145 = IC_50_ 15.85 μM; A549 = IC_50_ 86.62 μM	[58]
Baeckfrutone D (**127**)	*Baeckea frutescens*	**Cytotoxic effects**: HCT116 = IC_50_ 38.32 μM; Hela = IC_50_ 83.85 μM; DU145 = IC_50_ 6.46 μM; A549 = IC_50_ 76.47 μM	[58]
Baeckfrutone F (**129**)	*Baeckea frutescens*	**Cytotoxic effects**: HCT116 = IC_50_ 39.5 μM; DU145 = IC_50_ 80.72 μM; A549 = IC_50_ 15.61 μM; **Anti-inflammatory effects**: 74.4%	[58]
Baeckfrutone G (**130**)	*Baeckea frutescens*	**Cytotoxic effects**: HCT116 = IC_50_ 49.76 μM; Hela = IC_50_ 31.87 μM; DU145 = IC_50_ 17.40 μM; A549 = IC_50_ 62.64 μM; **Anti-inflammatory effects**: 75.3%	[58]
Baeckfrutone H (**131**)	*Baeckea frutescens*	**Cytotoxic effects**: HCT116 = IC_50_ 19.50 μM; Hela = IC_50_ 30.44 μM; DU145 = IC_50_ 25.14 μM; A549 = IC_50_ 82.75 μM; **Anti-inflammatory effects**: 55.1%	[58]
Baeckfrutone I (**132**)	*Baeckea frutescens*	**Cytotoxic effects**: HCT116 = IC_50_ 19.50 μM; Hela = IC_50_ 53.71 μM; DU145 = IC_50_ 26.11 μM; A549 = IC_50_ 84.13 μM; **Anti-inflammatory effects**: 75%	[58]
Baeckfrutone J (**133**)	*Baeckea frutescens*	**Cytotoxic effects**: HCT116 = IC_50_ 52.93 μM; DU145 = IC_50_ 4.04 μM; A549 = IC_50_ 79.45 μM	[58]
Baeckfrutone K (**134**)	*Baeckea frutescens*	**Cytotoxic effects**: HCT116 = IC_50_ 12.89 μM; DU145 = IC_50_ 77.06 μM; A549 = IC_50_ 80.11 μM	[58]
Baeckfrutone L (**135**)	*Baeckea frutescens*	**Cytotoxic effects**: HCT116 = IC_50_ 16.48 μM; Hela = IC_50_ 19.81 μM; DU145 = IC_50_ 10.0 μM; A549 = IC_50_ 88.81 μM	[58]
Hyperjaponol H (**143**)	*Hypericum japonicum*	**Antiviral effects**: EBV = EC_50_ 25.0 μM	[61]
Tomentosenol A (**144**)	*Rhodomyrtus tomentosa*	**Cytotoxic effects**: MCF-7 = IC_50_ 8.66 μM; NCI-H460 = IC_50_ 8.62 μM; SF-268 = IC_50_ 10.01 μM; HepG-2 = IC_50_ 9.44 μM	[61]
Myrtucommulone (**147**)	*Myrtus communis*	**Cytotoxic effects**: HepG2 = IC_50_ 4.39 μM; MDA-MB-231 = IC_50_ 19.92 μM	[62]
Myrtucommulone (**148**)	*Myrtus communis*	**Cytotoxic effects**: HepG2 = IC_50_ 40.7 μM; MDA-MB-231 = IC_50_ 40.0 μM	[62]
Frutescone A (**151**)	*Baeckea frutescens*	**Cytotoxic effects**: Caco-2 = IC_50_ 8.08; A549 = IC_50_ 20.07 µM	[64]
Frutescone B (**152**)	*Baeckea frutescens*	**Cytotoxic effects**: Caco-2 = IC_50_ 23.25 µM; A549 = IC_50_ 41.33 µM	[64]
Frutescone C (**153**)	*Baeckea frutescens*	**Cytotoxic effects**: Caco-2 = IC_50_ 14.83 µM; A549 = IC_50_ 27.74 µM	[64]
Frutescone D (**154**)	*Baeckea frutescens*	**Cytotoxic effects**: Caco-2 = IC_50_ 10.20 µM; A549 = IC_50_ 26.25 µM	[64]
Frutescone E (**155**)	*Baeckea frutescens*	**Cytotoxic effects**: Caco-2 = IC_50_ 7.96 µM; A549 = IC_50_ 5.55 µM	[64]
Frutescone F (**156**)	*Baeckea frutescens*	**Cytotoxic effects**: Caco-2 = IC_50_ 16.51 µM; A549 = IC_50_ 39.02 µM	[64]
(±)-Frutescone G (**157**)	*Baeckea frutescens*	**Cytotoxic effects**: Caco-2 = IC_50_ 14.31 µM; A549 = IC_50_ 25.71 µM	[64]
Callisalignene G (**174**)	*Callistemon salignus*	**Cytotoxic effects**: HCT116 = IC_50_ 8.51 μM; A549 = IC_50_ 12.85 μM	[67]
Callisalignene H (**175**)	*Callistemon salignus*	**Cytotoxic effects**: HCT116 = IC_50_ 9.12 μM	[67]
Callisalignene I (**176**)	*Callistemon salignus*	**Cytotoxic effects**: HCT116 = IC_50_ 16.33 μM; A549 = IC_50_ 10.03 μM	[67]
Frutescone I (**178**)	*Baeckea frutescens*	**Anti-inflammatory effects**: NO production = IC_50_ 18.75 μM	[68]
Frutescone L (**179**)	*Baeckea frutescens*	**Anti-inflammatory effects**: NO production = IC_50_ 30.54 μM	[68]
Frutescone M (**180**)	*Baeckea frutescens*	**Anti-inflammatory effects**: NO production = IC_50_ 15.17 μM	[68]
(±)-Compound (**181**)	*Baeckea frutescens*	**Anti-inflammatory effects**: NO production = IC_50_ 1.80 μM	[68]
Compound (**182**)	*Baeckea frutescens*	**Anti-inflammatory effects**: NO production = IC_50_ 0.36 μM	[68]
Compound (**183**)	*Baeckea frutescens*	**Anti-inflammatory effects**: NO production = IC_50_ 3.70 μM	[68]
(±)-Compound (**184**)	*Baeckea frutescens*	**Anti-inflammatory effects**: NO production = IC_50_ 2.07 μM	[68]
(±)-Compound (**185**)	*Baeckea frutescens*	**Anti-inflammatory effects**: NO production = IC_50_ 6.50 μM	[68]
Baefrutone A (**188**)	*Baeckea frutescens*	**Anti-inflammatory effects**: NO Production = IC_50_ 9.15 μM	[69]
Baefrutone B (**189**)	*Baeckea frutescens*	**Anti-inflammatory effects**: NO Production = IC_50_ 17.73 μM	[69]
Baefrutone C (**190**)	*Baeckea frutescens*	**Anti-inflammatory effects**: NO Production = IC_50_ 11.62 μM	[69]
Baefrutone D (**191**)	*Baeckea frutescens*	**Anti-inflammatory effects**: NO Production = IC_50_ 18.04 μM	[69]
hyperjaponols A (**194a**)	*Hypericum japonicum*	**Antiviral effects**: EBV = EC_50_ 10.33 μM	[70]
Hyperjaponol B (**195a**)	*Hypericum japonicum*	**Antiviral effects**: EBV = EC_50_ 0.57 μM	[70]
Hyperjaponol B (**195b**)	*Hypericum japonicum*	**Antiviral effects**: EBV = EC_50_ 6.60 μM	[70]
Hyperjaponol D (**197**)	*Hypericum japonicum*	**Antiviral effects**: EBV = EC_50_ 0.49 μM	[70]

## 5. Alklaoid-Based Meroterpenoids

### Phenazine- and Phyridine-Based Meroterpenoids

**Figure 26:**
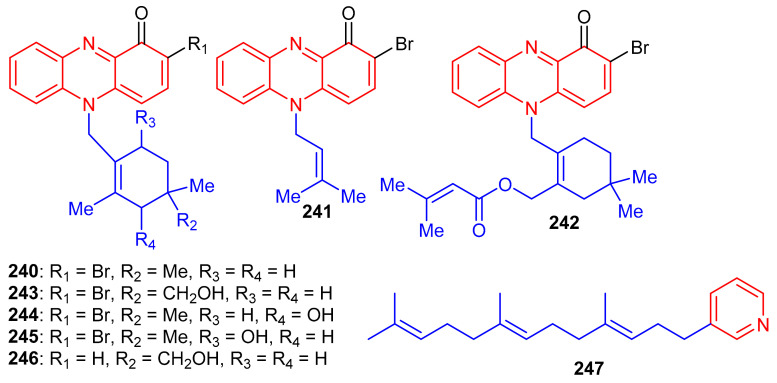
Structures of alkaloid-based meroterpenoids **240**–**247**.

Phenazine-derived meroterpenoids, viz., marinocyanins A–F (**240**–**245**) (Figure 26) along with lavanducyanin (**246**) were produced by the marine *Actinomycete* strains. Compounds **240**–**245** are unique secondary metabolites comprising the bromo-phenazinone nucleus supplemented by *N*-isoprenoid moieties or a cyclolavandulyl ring in their structures [79]. Lavanducyanin (**246**) was re-isolated from *Streptomyces* sp. as a testosterone 5α-reductase inhibitor and was named WS-9659A Quite recently Kohatsu et al. [80] reported the total synthesis of lavanducyanin (**246**). Marinocyanin A (**240**) has been reported to be a potent antibiotic, since it potentially inhibited (MIC = 0.95 μM) the growth of amphotericin-resistant *Candida albicans* in vitro, while the other test compounds were reported as only weak inhibitors (Table 3). In addition, marinocyanins A (**240**) and B (**241**) illustrated significant in vitro cytotoxic effects towards human colon carcinoma (HCT-116: **240**: IC_50_: 0.049 μM; **170**: IC_50_: 0.029 μM). SAR studies showed that the cyclic structure of the terpenoidal part (cyclolavandulyl ring) plays a significant role in the antifungal activity, and that the halogen plays no particular role in the activity [79]. The standard drugs used in these assays were vancomycin (MIC = 0.27 µM) for *S. aureus* and amphotericin B (MIC = 0.084 µM) for *C. albicans*. Zhang et al. [81] isolated an unusual C21 pyridine bearing meroterpenoid **247** from the sponge *Cacospongia* sp.

## 6. Sesquiterpene-Based Meroterpenoids

**Figure 27:**
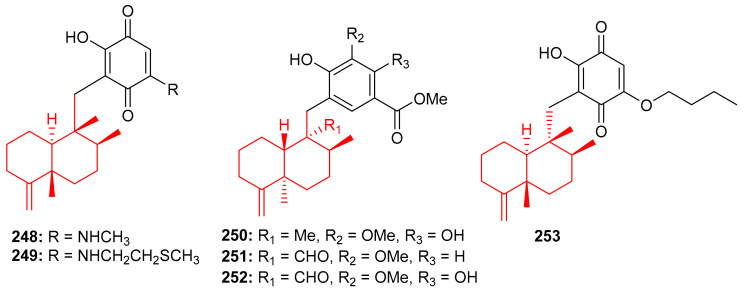
Structures of sesquiterpene-based meroterpenoids **248**–**253**.

The Vietnamian marine sponge *Spongia* sp. produces a range of meroterpenoids viz., langcoquinone A (**248**) and B (**249**) (Figure 27). On the other hand, compounds **248** and **249** were inactive against *K. pneumoniae* and *E. coli*, compared to the positive control Kanamycin ((MIC = 6.25 and 12.5 µM, respectively) [82].

In another investigation, Nguyen et al. [83] further isolated sesquiterpene-based meroterpenoids, langconols A–C (**250**–**252**) (Figure 27) and langcoquinone C (**253**) from the same sponge viz., *Spongia* sp. Furthermore, compounds **250**–**252** bear the 4,9-friedodrimane skeleton along with phenolic functionality while langcoquinone C (**253**) has an hydroxyquinone instead of the phenolic group. Compound **253** exhibited significant inhibitory activity (MIC = 6.25 µM) against *B. subtilis* and *S. aureus*, with the same potential as mentioned above for the reference drug ampicillin, whereas, compounds **250** and **253** only inhibited the growth of *B. subtilis* with MICs of 12.5 and 25.0 μM, respectively. Compound **250** has good potential to be an antibacterial and non-toxic agent and thus offers itself as a strong candidate to be studied for the development of a potentially new antibiotic [83].

**Figure 28:**
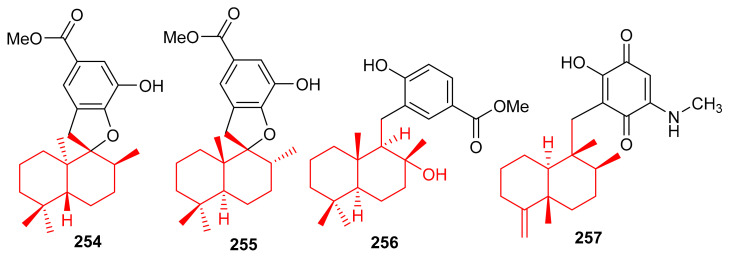
Structures of sesquiterpene-based meroterpenoids **254**–**257**.

The marine sponge *Dysidea* sp. produces the sesquiterpene-based meroterpenoids dysidphenols A–C (**254**–**256**), along with smenospongimine (**257**), (Figure 28) all of which were characterized by spectroscopic analyses and ECD calculations [84]. Moreover compounds **254**–**256** all comprise a drimane-type sesquiterpene unit attached to a phenolic entity through either an oxaspiro center or methylene linkage. On the other hand, compound **267** comprises the 4,9-friedodrimane skeleton attached to hydroxybenzoquinone moieties. Compounds **254** and **256** were weakly active against *E. coli*, *B. subtilis* and *S. aureus*. However, the other test compounds **257** was found to be more potent against these three bacterial species with MIC values between 3.1 and 12.5 µg/mL (Table 4).

**Figure 29:**
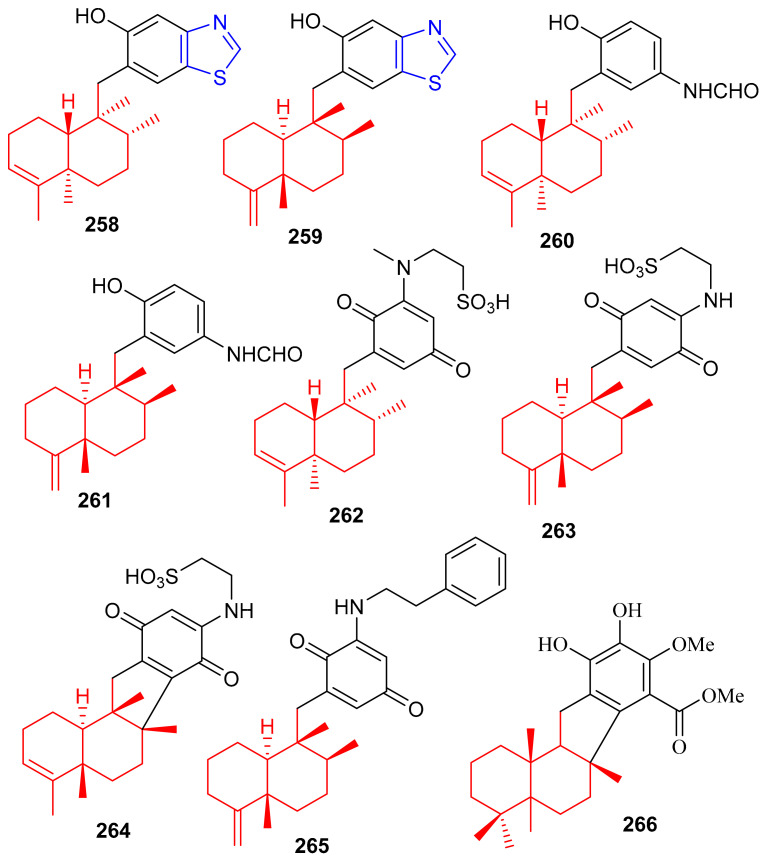
Structures of sesquiterpene-based meroterpenoids **258**–**266**.

The marine sponge *Dactylospongia* sp. has been reported to produce several meroterpenoids including dactylospongins A–D (**258**–**261**), melemeleones C–E (**262**–**264**), dysidaminone N (**265**) and 19-*O*-methylpelorol (**266**). Compounds **258**–**265** (Figure 29) have a sesquiterpene moiety attached to either a benzothiazole, phenolic, or benzoquinone core through a C-C bond [85]. An interesting feature of meroterpenoids **258** and **259** is that they comprise a unique thiazole ring which biogenetically, could be derived from cysteine [86].

**Figure 30:**
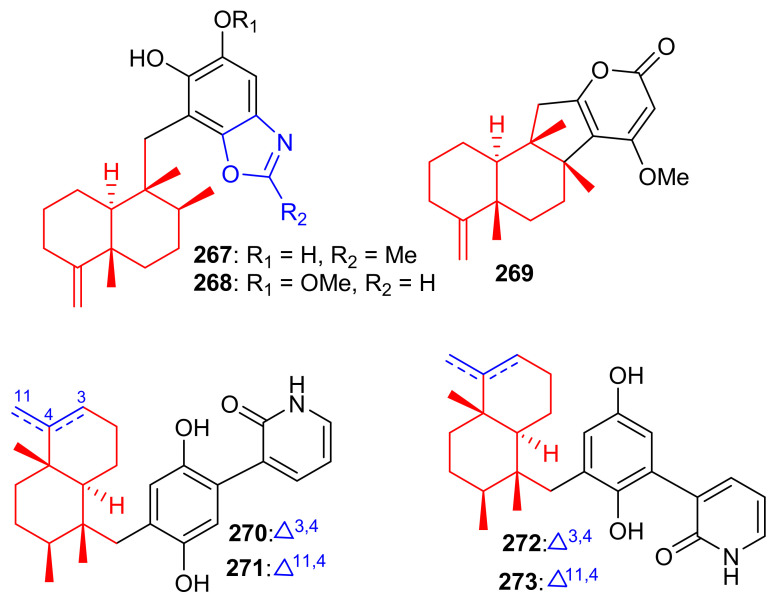
Structures of sesquiterpene-based meroterpenoids **267**–**273**.

Among other meroterpenoid inhibitors of PTP1B, nakijinol G (**267**) (Figure 30) inhibited the activity of this enzyme with an IC_50_ value of 4.8 μM (Table 4). However, the other metabolites, nakijinol F (**268**), hyrtiolacton A (**269**) all isolated from the same marine sponge *Hyrtios* sp. were inactive. Moreover, none of these compounds were active towards HepG2, RPMI-8226, HeLa, and HL-60 cancer cells. Compounds **267** and **268** have a sesquiterpene coupled to a benzoxazole moiety while in compound **269** the benzoxazole ring is replaced by an α-pyrone and benzoquinone unit respectively [87]. Another sponge, *Dysidea villosa* also produces some unusual meroterpenoids described as dysivillosins A–D (**270**–**273**), which all inhibited the release of β-hexosaminidase with IC_50_ ranging from 8.2 to 19.9 µM (Table 4). In addition, compounds **270**–**273** exert a positive inhibitory effect on LTB-4 and IL-4 and compound **270** potentially inhibited the activation of Syk. It may thus be concluded that this meroterpenoid could potentially be a new chemotherapeutic scaffold targeting Syk-associated allergies [88]. Dysivillosins A–D (**270**–**273**) (Figure 30) are meroterpenes bearing a terpene-polyketide-pyridine system and this type of combination is very rare among meroterpenoids. Moreover, meroterpenoids **270**–**273** have a 2-pyridone core which could be produced biogenetically from L-lysine through amidation, decarboxylation, and dehydrogenation reactions [89].

**Figure 31:**
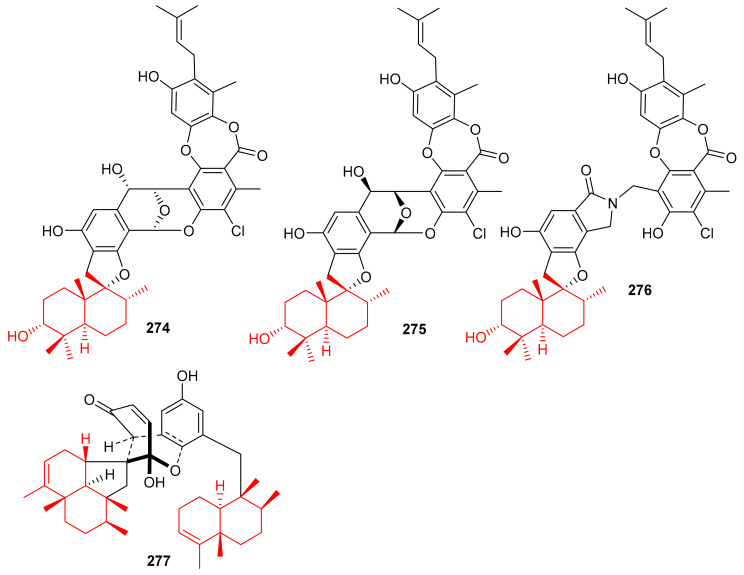
Structures of sesquiterpene-based meroterpenoids **274**–**277**.

Chartarolides A–C (**274**–**276**) (Figure 31), the secondary metabolites of the sponge *Niphates recondite,* were tested for their cytotoxic properties against HCT-116, BGC-823, HepG2, A2780 NCI-H1650, and MCF7 cancer cells. Compound **274** displayed the most potent effects with IC_50_ values ranging from 1.3 to 1.9 μM, followed by compound **275** (IC_50_ = 1.6–4.8 μM) (Table 4), while compound **276** has been reported to be the least active with IC_50_ values in the range of 5.4–12.5 μM (Table 4) [90]. However, the activities of these metabolites are lower than the reference drug taxol, which displayed IC_50_ values of 0.001 to 0.07 against these cell lines. In addition, compounds **274**–**276** have also been reported as inhibitors (IC_50_ = 2.6–21 μM) of FGFR3, IGF1R and PDGFRb [90], which is lower than the activity of the positive control satratoxin H (IC_50_ = 0.05 µM). Another marine sponge, *Dysidea arenaria* produces dysiarenone (**277**) and this compound displayed inhibitory activities towards COX-2 expression with an IC_50_ value of 6.4 μM. Compound **277** is a dimeric C-21 meroterpenoid featuring a unique 2-oxaspiro(bicyclo[3.3.1]nonane-9,1′-cyclopentane) carbon skeleton [91]. This compound reduced the production of PGE2 with IC_50_: 6.4 μM and was ~10 times more potent than that of the positive control avarol [91].

**Figure 32:**
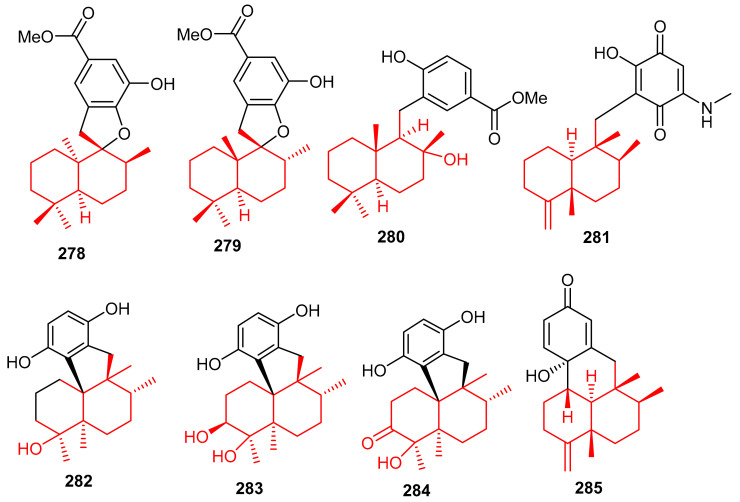
Structures of sesquiterpene-based meroterpenoids **278**–**285**.

Meroterpenoids **278**–**281** (Figure 32) were produced by the sponge *Dysidea* sp. [84] and their absolute configuration were determined via CD and ECD calculations [92]. In another report, *Dysidea* sp. also produced meroterpenoids, dysiherbols A–C (**282**–**284**) featuring a 6/6/5/6-fused core and dysideanone E (**285**). Moreover, compounds **282**–**284** illustrated potent NF-κB inhibition with IC_50_ ranging from 0.49–6.4 µM. Notably, compound **282** was potent towards myeloma cancer (NCI H-929: IC_50_: 0.58 µM) as well as a potent NF-κB inhibitor with IC_50_ = 0.49 µM) [93].

**Figure 33:**
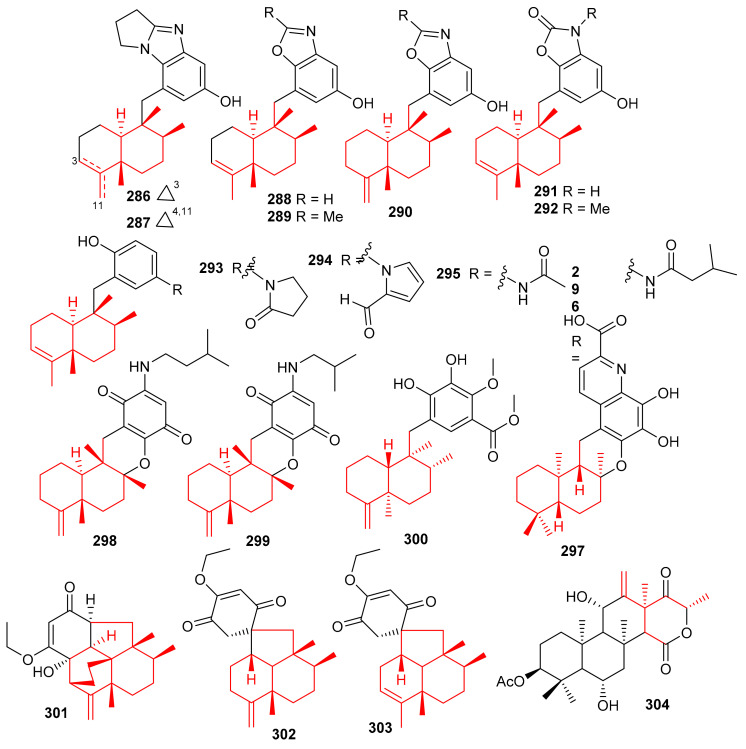
Structures of sesquiterpene-based meroterpenoids **286**–**304**.

Cinerols A–K (**286**–**296**) (Figure 33) were produced by the sponge *Dysidea cinerea* which was collected from the China Sea. Compounds **286**–**288** illustrated good PTP1B inhibitory effects with IC_50_ values of 3.8–8.8 μM. On the other hand, only compound **291** was active towards the SHP-1 enzyme with IC_50_: of 2.7 μM [94]. Saccharoquinoline (**297**) was isolated from the bacterium *Saccharomonospora* sp. and featured a drimane-type sesquiterpene unit. Saccharoquinoline (**286**) exhibited good cytotoxicity towards HCT-116 cancer [95]. Three sesquiterpene based meroterpenes **298**–**300** were isolated from the sponge *Dactylospongia elegans* and compounds **300** illustrated cytotoxic effects towards SW1990, DU145, PANC-1, and Huh7 with IC_50_ values ranging from 2.3–37.8 µM [96]. Septosones A–C (**301**–**303**) were isolated from the sponge *Dysidea septosa* and septosone A (**301**) displayed good in vivo anti-inflammatory effects [97]. Terretonin N (**304**) (Figure 33) isolated from *Nocardiopsis* sp. illustrated a 15 mm of zone of inhibition towards *Staphylococcus warneri*, which has been observed to be even higher than the reference drug, gentamycin (14 mm) [98].

## 7. Chromane/Chromene and Flavone Derived Meroterpenoids

**Figure 34:**
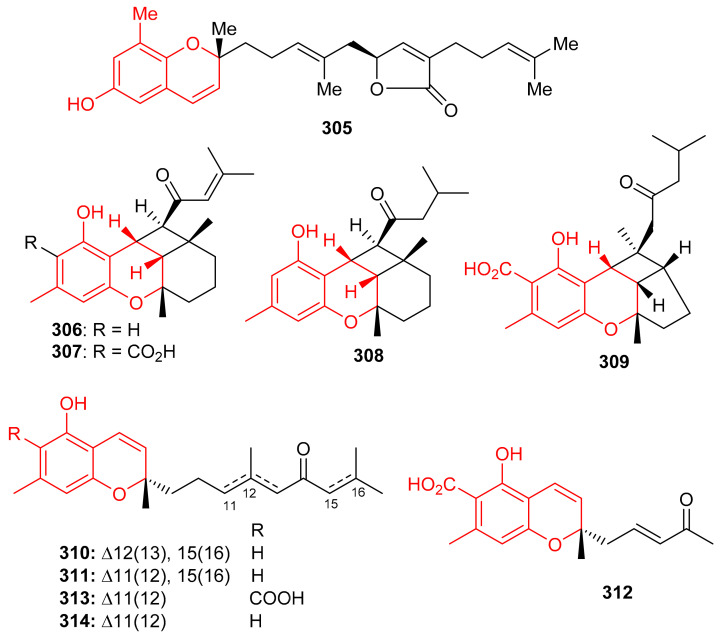
Structures of chromane/chromene derived meroterpenoids **305**–**314**.

Among other metabolites, the chromene-derived meroterpenoid with an additional furan ring within a prenyl moiety, tuberatolide B (**305**) (Figure 34), was initially reported from *Botryllus tuberatus* [99] and later from *Sargassum macrocarpum* [100]. This diastereomeric meroterpenoid is reported to display anticancer activity since it inhibits lung cancers (H1299 and A549), breast cancers (MDA-MB-453, MDA-MB-231, and MCF7), colon cancers (CT26, HCT116, and SW620), cervical cancer (HeLa), and prostate cancers (DU145 and PC3) [100]. The mechanistic study revealed that compound **305** inhibits the growth of cancer cells by the production ROS in HCT116, A549, and MDA-MB-231, cells. It also increases DNA damage by the formation of γH2AX foci and or the phosphorylation of Chk2 and H2AX, which proteins are generally associated with DNA damage [100].

Chromane/chromene meroterpenoids (CMs), the rubiginosins A–G (**306**–**312**) (Figure 34) and anthopogochromenes A (**313**) and B (**314**), were reported from *Rhododendron rubiginosum* [101]. In addition to spectroscopic techniques, their absolute structures were established by making use of the chromane/chromene helicity rule, X-ray crystallography and CD analysis. All these compounds were evaluated for their cytotoxic effects towards A549, HCT116, SK-HEP-1, and HL-60 (Table 4). Compound **310** was the most active against all cell lines with IC_50_ values of 10.91, 13.89, 11.71 and 7.40 μM, respectively and then followed compounds **306**, **308** and **314**. The other tested metabolites are reported to be inactive [101]. Doxorubicin (IC_50_ = 0.01–0.2 μM) was used as positive control in this study. Over 20 CMs have been reported from the genus *Rhododendron* bearing a cannabinoid-like and orcinol core. Moreover, *Rhododendron* CM was also designated as a cannabicyclol (CBL)-type or cannabichromene (CBC)-type [102]. Interestingly CBC/CBL-type natural products having an orcinoid skeleton are rare in *Cannabis* and are mostly reported from *Rhododendron species* [102].

**Figure 35:**
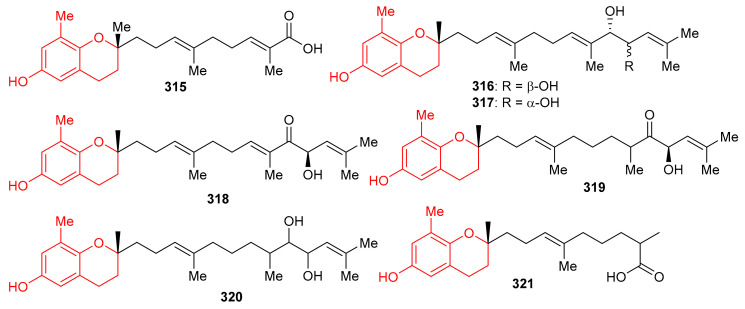
Structures of chromane/chromene derived meroterpenoids **315**–**321**.

*Sargassum siliquastrum* produced a small library of the meroterpenoids isopolycerasoidol (**315**), sargachromanols D (**316**), E (**317**), G (**318**), I (**319**), S (**320**), and T (**321**) (Figure 35) and all were evaluated for their antioxidant effects. Compound **315** was the most active in DPPH and ABTS antioxidant assays with EC_50_ values of 8.23 and 2.33 μM, respectively [103]. Compounds **316**–**318** were weakly active against the DPPH free radical, but induced significant inhibition (EC_50_: 4.0 to 4.8 μM, Table 4) against the ABTS free radical. On the other hand, compounds **316** and **317** are only weakly active in DPPH and ABTS antioxidant assays with EC_50_ values ranging from 15.7 to 57.0 μM. The structure and activity variation of compounds **316**–**318** suggest that the hydroxyl group at C-13 in the prenyl moiety can be the activity determining factor, since compounds **316** and **317** have the hydroxyl group at C-12, while compounds **318** and **319** have a corresponding keto function. Other literature results show that the chromene nucleus is an important group for antioxidant activities [103].

**Figure 36:**
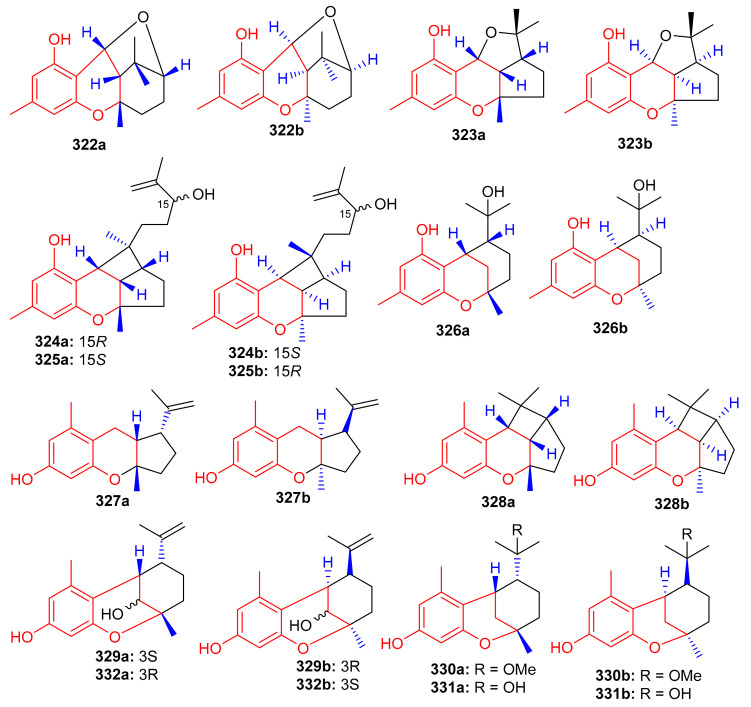
Structures of chromane/chromene derived meroterpenoids **322**–**332**.

Another medicinal plant, *Rhododendron capitatum* produces enantiomeric pairs of meroterpenoids, the (±)-rhodonoids C–G (**322**–**326**) (Figure 36). These compounds existed as racemates and were subsequently purified via chiral HPLC. Moreover, only **322a** inhibited HSV-1 with an IC_50_ value of 80.6 µM. Compounds **322a** and **322b** featured the unusual 6/6/6/5 tetracyclic ring core while compounds **323a** and **323b** bore the rather rare 6/6/5/5 tetracyclic ring system [104]. Another *Rhododendron* sp., viz., *R. nyingchiense* interestingly, also produced enantiomeric pairs of the meromonoterpenoids **327a**,**b**–**332a**,**b** and all racemic mixtures were separated by chiral-phase HPLC. These compounds possess PTP1B inhibition with IC_50_ values ranging from 29 to 61 μM. Compounds **327a**,**b** feature a quite rare 6/6/5 tricyclic ring core [105].

**Figure 37:**
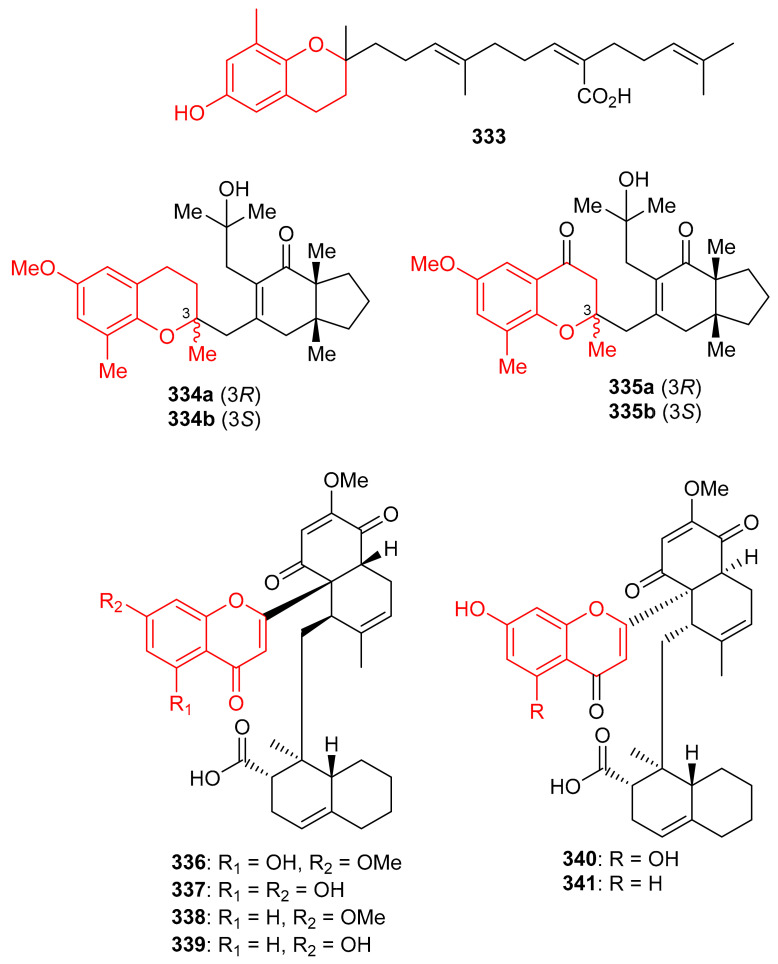
Structures of chromane/chromene derived meroterpenoids **333**–**341**.

Sargachromenol (**333**) (Figure 37) was produced by *Sargassum serratifolium* and inhibited BChE and BACE1 with values for IC_50_: 9.4 and 7.0 μM respectively [106], while the reference compounds used were berberine (IC_50_ = 9.4 µM) and quercetin (IC_50_ = 5.6 µM) respectively. The alga *Cystoseira baccata* produced racemic mixtures of two meroterpenoids, tetraprenyltoluquinol (**334a**,**b**), and tetraprenyltoluquinone (**335a**,**b**). The in vitro anti-leishmanial study demonstrated that compound mixture **334a**,**b** exhibited reasonable effects towards *Leishmania infantum* with IC_50_: 44.9 μM, whereas compound mixture **335a**,**b** was found to be a weak inhibitor with IC_50_ of 94.4 μM. In an SAR study, it was determined that the C-1 ketone decreases the anti-leishmanial effects since the difference between these two compounds viz., **334** and **335** is the keto group [107].

Diplomeroterpenoids A–F (**336**–**341**) (Figure 37), were isolated from the roots of *Mimosa diplotricha* and featured the diterpenoid unit and chromen-4-one framework. Compounds **336**–**338** and **340** inhibited protein farnesyl transferase (PFTase) with an IC_50_ ranging from 5.0 to 8.5 μM [108]. Activity of the reference inhibitor FTase inhibitor II is reported as IC_50_ = 0.1 µM. Additionally, diplomeroterpenoid A (**336**) is also reported to inhibit the growth of HepG2 cancer cells with a GI_50_: 8.6 μM.

**Figure 38:**
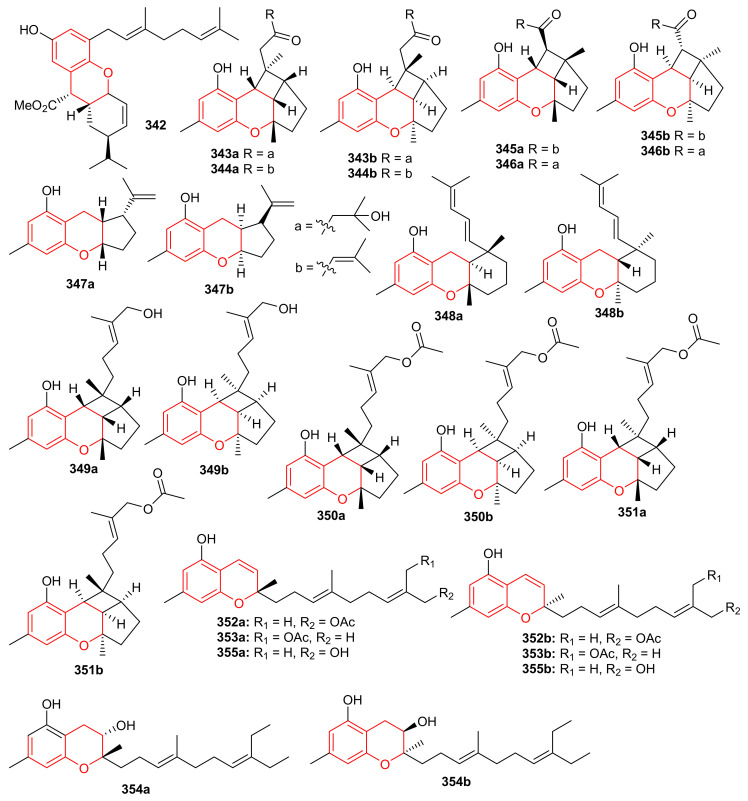
Structures of chromane/chromene derived meroterpenoids **342**–**354**.

Glabralides C (**342**) was isolated from *Sarcandra glabra* [109]. Five pure meroterpenoid enantiomers (**343a**/**343b**–**347a**/**347b**) were produced by *Rhododendron fastigiatum*. Moreover, meroterpenoids **344a**/**344b**, **345a**/**345b**, demonstrated PTP1B inhibitory effects with IC_50_ ranging from 40.9 to 47.0 [110]. (+)-/(−)-Anthoponoids A–G, (**348**–**354**) (Figure 38), and (+)/(−)-daurichromene D (**355**) were isolated from *Rhododendron anthopogonoides* and (+)-anthoponoid E (**352a**), (−)-anthoponoid G (**354b**), exhibited potent anti-inflammatory effects in RAW 264.7 macrophages [111].

**Figure 39:**
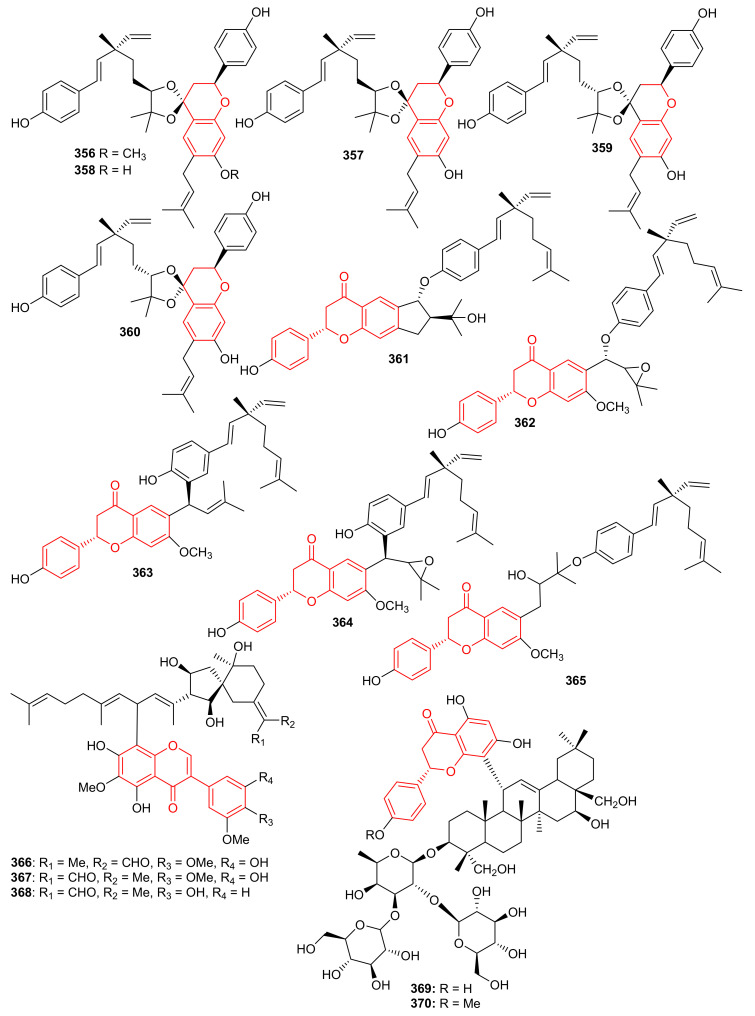
Structures of chromane/chromene and flavone derived meroterpenoids **356**–**370**.

Psocorylins A–J (**356**–**365**) (Figure 39) were produced by *Psoralea corylifolia* and evaluated for their cytotoxic effects towards HepG2, NCI-N87, HeLa, HCT-116, and B16-F10. Meroterpene **357** displayed remarkable cytotoxic effects towards these five cell lines with IC_50_: <10 μM. On the other hand, compounds **358**–**360** also illustrated significant effects towards HepG2 and NCI-N87 with IC_50_: < 10 μM, which demonstrated selectivity towards these two cancer cells. Compound **356** illustrated no cytotoxicity but indicated the decreased cytotoxic effects due to the presence of a methoxy group at C-7. Notably, compound **361** displayed potent cytotoxic effects towards HepG2, NCI-N87, HeLa, and HCT-116 with IC_50_ values from 1.82 to 5.74 μM, respectively [112]. Belamcandanins A–C (**366**–**368**), were isolated from *Belamcanda chinensis* [113]. The highly functionalized, flavonoid-derived triterpene saponin meroterpenoids, clinoposides G (**369**) and H (**370**) (Figure 39) were isolated from *Clinopodium chinense*. Both compounds featured a triterpenoid core linked to a flavonoid framework via a C-C bond. The cardioprotective effects of compounds **369** and **370** were evaluated and both compounds illustrated protective effects towards anoxia/reoxygenation(A/R)-induced injury in H9c2 cells [114].

## 8. Quinone-Based Meroterpenoids

**Figure 40:**
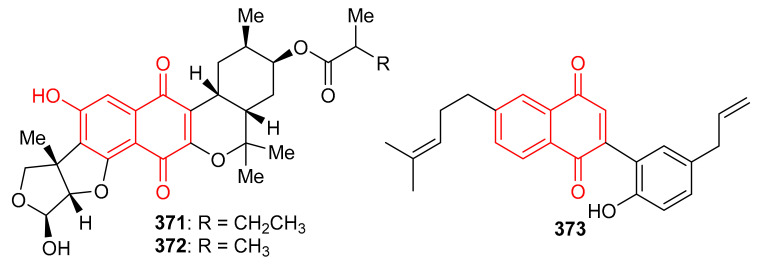
Structures of quinone-based meroterpenoids **371**–**373**.

*Streptomyces* sp. produced naphthoquinone based meroterpenoids described as naphthablins B (**371**) and C (**372**) (Figure 40) and the absolute structures of these compounds were established by ECD spectra and the TDDFT approach. Compounds **371** and **372** were reported to be weakly active towards HeLa cells (19 to 32%) [115]. Magterpenoid C (**373**) was reported from *Magnolia officinalis* var. and illustrated significant PTP1B inhibition with an IC_50_ value of 0.81 μM [116].

**Figure 41:**
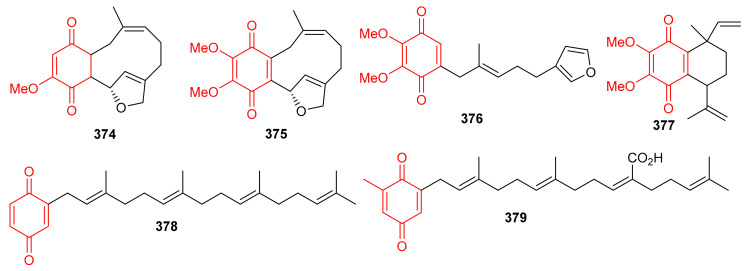
Structures of quinone-based meroterpenoids **374**–**379**.

In 2018, arnebinone B (**374**), compound **375**, arnebifuranone (**376**), and arnebinone (**377**) (Figure 41) were isolated from *Arnebia euchroma* and tested against various liver cancer cell lines viz., SMMC-7721, HepG2/ADM HepG2, and QGY-7703. Compound **375** was the most active among all the tested cell lines with IC_50_ values ranging from 3.43 to 11.31 μM, while compound **374** had IC_50_ values ranging from 9.6 to 18.7 μM. These activites are reported even higher than the activity of the reference drug Cisplatin (IC_50_ = 5.66–27.96 μM). On the other hand, the activities of compounds **376** and **377** were not that impressive (Table 5) [117]. Meroterpneoid **375** was also reported but with a different name viz., JNU-144, from *Lithospermum erythrorhizon* as a new compound in the same year (2018). The present study seems interesting in the sense that compound **375** has been reported to suppress cell viability and proliferation in hepatoma cells [118]. Toluquinol-derived meroterpenoid (**378**) bearing a tetraprenyl moiety, was isolated from the *Carteriospongia* sp. and was subsequently shown to trigger MMP disruption and apoptosis in lymphoma (U937 and Sup-T1 cells), leukemia (Molt 4 and HL60 cells), oral (Ca9-22 and Cal-27 cells), breast (T-47D cells) with IC_50_ values ranging from 0.33 to 1.06 μg/mL [119]. Doxorubicin, a positive control displayed IC_50_ values in the range of 0.1–2.47 μg/mL, which revealed that the test compounds are equally active against the cell lines. Sargaquinoic acid (**379**) was reported from *Sargassum serratifolium* and inhibited the activity of AChE, BChE and BACE1, (Table 5) with IC_50_ values of 69.3, 10.5, and 12.1 μM respectively [106].

**Figure 42:**
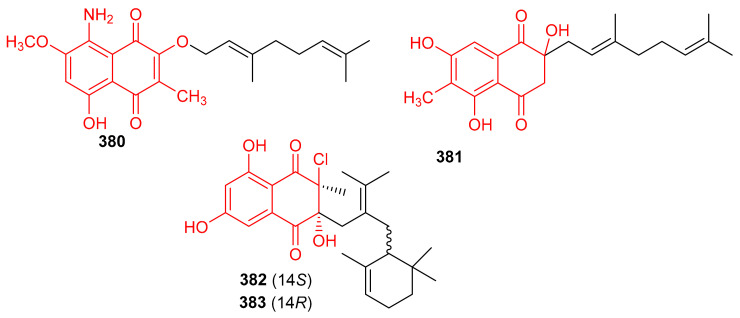
Structures of quinone-based meroterpenoids **380**–**383**.

Two naphthoquinone based meroterpenes named Flaviogeranin B (**380**) and Flaviogeranin D (**381**) (Figure 42) were produced by *Streptomyces* sp. Notably, compound **381** illustrated significant antibacterial effects towards *Mycobacterium smegmatis* and *Staphylococcus aureus* with MIC values ranging from 5.2 to 9.2 µg/mL. In addition, compound **381** also possessed potent cytotoxic effects towards HeLa and A549 with IC_50_: 0.4 and 0.6 µM, respectively [120]. In another report *Streptomyces* sp. also produce merochlorins E (**382**) and F (**383**) which exhibited significant antibacterial effects towards *B. subtilis*, *S. aureus*, *K. rhizophila* with MIC ranging from 1–2 μg/mL [121].

## 9. Miscellaneous

**Figure 43:**
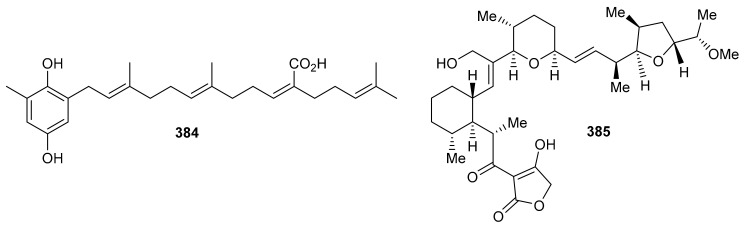
Structures of miscellaneous meroterpenoids **384** and **385**.

Sargahydroquinoic acid (**384**) (Figure 43) was isolated from *Sargassum serratifolium* and inhibited BChE and BACE1 with values of IC_50_: 15.2 and 4.4 μM respectively [106]. Compared to the reference drugs berberine (IC_50_ = 9.4 µM) and quercetin (IC_50_ = 5.6 µM), respectively, the activities of these natural products are significant. Martucci et al. studied anticancer properties of tetronasin (**385**) which was obtained from *Streptomyces* sp. CP26-58 by HRLCMS. Tetronasin (**385**) killed the HeLa cells with an IC_50_ value of 0.23 μM [115].

**Figure 44:**
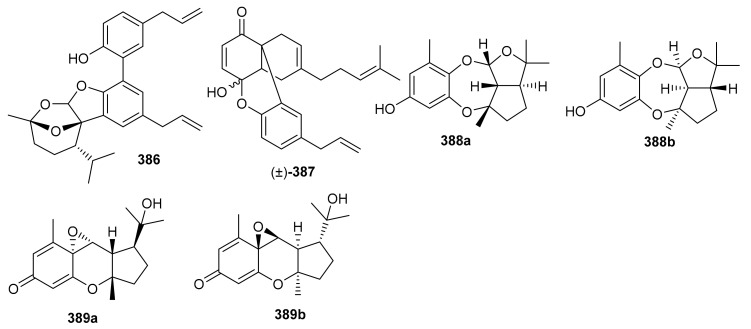
Structures of miscellaneous meroterpenoids **386**–**389**.

Magterpenoids A (**386**) and B (**387**) (Figure 44) were purified from the bark extract of *Magnolia officinalis* and tested for PTP1B inhibitory activities. Compound **386** displayed a significant inhibition of the enzyme with an IC_50_ value of 1.44 μM, which is higher than the positive control drugs donepezil (45.3% and 46.2%). Moreover magterpenoid A (**386**) featured an interesting 4,6,11-trioxatricyclo[5.3.1.01,5]undecane skeleton while compound **387** had a 6/6/6/6 tetracyclic core [116]. Nyingchinoids A (**388a**,**b**) and B (**389a**,**b**) were separated from the plant *Rhododendron nyingchiense*. Metabolites **388a**/**b** and **389a**,**b** featured 6/7/5/5 and 6/6/3/5 heterocyclic ring frameworks respectively. Notably, compounds **388a**/**b** and **389a**,**b** illustrated PTP1B effects with IC_50_ values of 43.6 and 38.1 µM, respectively. In this assay, oleanolic acid was used as positive control, which showed an IC_50_ value of 2.5 μM [105].

**Figure 45:**
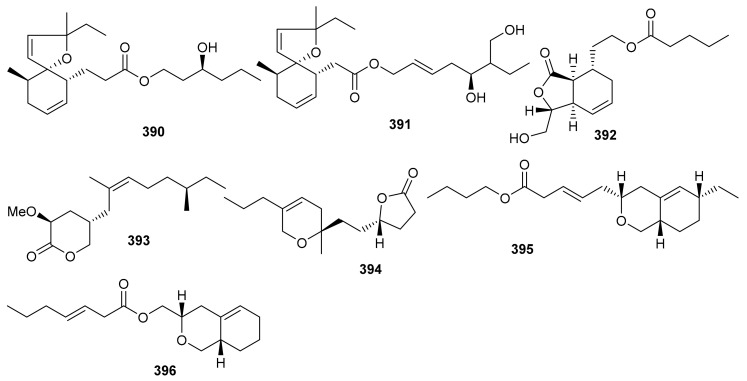
Structures of miscellaneous meroterpenoids **390**–**396**.

*O*-Spirocyclic ether analogs viz., **390** and **391** along with butanolide (**392**) (Figure 45) were obtained from *Villorita cyprinoides*, which is a traditional seafood in the coastal regions of the Arabian Sea. Meroterpenoid **391** illustrated activity towards COX-1 (IC_50_: 0.86 mg/mL) and COX-2 (IC_50_: 0.65 mg/mL) enzymes, followed by compound **390** (COX-1: IC_50_: 0.94 mg/mL; COX-2: IC_50_: 0.70 mg/mL) [122]. On the other hand, compound **392** was also found to be active towards COX-1 (IC_50_: 0.91 mg/mL) but slightly less active than COX-2 (IC_50_: 0.74 mg/mL) when compared to compound **390** and **391**. Further biological studies revealed that compounds **390**–**392** demonstrated 5-LOX inhibitory effects (IC_50_ = 0.77, 0.75, 0.80 mg/mL, respectively and their effects were higher than the standard ibuprofen (IC_50_ = 0.96 mg/mL). In addition, these meroterpenoids illustrated more potent selectivity indices (SI: IC_50_: 1.23–1.34) than ibuprofen (SI: IC_50_: 0.63). Meroterpenoids **390** (IC_50_: 0.59 mg/mL) and **391** (IC_50_: 0.54 mg/mL) possess potent DPPH antioxidant effects which are higher than the standard α-tocopherol (IC_50_ = 0.65 mg/mL). Of note, compound **392** illustrated slightly less activity (IC_50_ = 0.69 mg/mL) than the previous two compounds [122].

*Villorita cyprinoides* also produces four meroterpenoids viz., **393**–**396** (Figure 45) which were evaluated for various biological effects. Meroterpenoids **393**–**396** illustrated potent effects towards COX-1 and COX-2 in which the IC_50_: ranged from 0.84 to 1.09 mg/mL [123], while the positive control ibuprofen exhibited IC_50_ values of 0.05 and 0.08 mg/mL, respectively. Additionally, these compounds possess anti-COX-1/anti-COX-2 activity with IC_50_: ranging from 1.12 to 1.22 mg/mL (Table 5). On the other hand, meroterpenoids **393**–**396** also display significant anti-5-LOX potential effects with IC_50_ values ranging from 0.76 to 0.98 mg/mL with the effects of **393** (IC_50_ = 0.92 mg/mL) and **394** (IC_50_ = 0.76 mg/mL) being higher than the standard ibuprofen (IC_50_ = 0.96 mg/mL). Furthermore, meroterpenoids **393**–**396** also displayed DPPH antioxidant effects with IC_50_ values ranging from 0.63 to 0.79 mg/mL [123].

**Figure 46:**
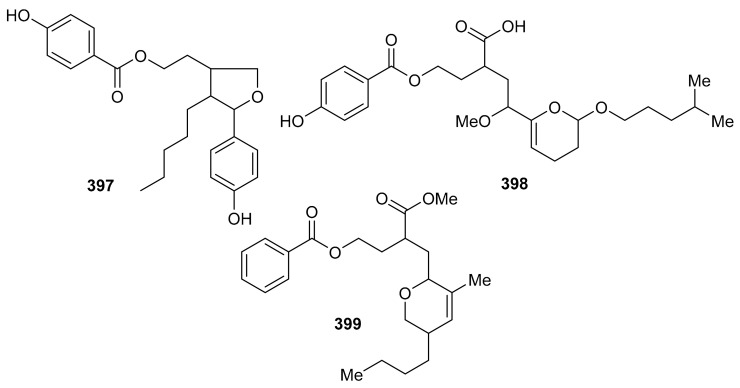
Structures of miscellaneous meroterpenoids **397**–**399**.

Erlotinib, the reference drug in this study, showed IC_50_ values of 4.5 and 7.66 µM, respectively. Meroterpenoids, 2-[tetrahydro-5-(4-hydroxyphenyl)-4-pentylfuran-3-yl]-ethyl-4-hydroxybenzoate (**397**), 2-2-[(4-hydroxybenzoyl)-oxy]-ethyl-4-methoxy-4-2-[(4-methylpentyl)oxy]-3,4-dihydro-2*H*-6-pyranylbutanoic acid (**398**) and 3-[(5-butyl-3-methyl-5,6-dihydro-2*H*-pyran-2-yl)-methyl]-4-methoxy-4-oxobutyl benzoate (**399**) (Figure 46) were reported from the alga *Hypnea musciformis* and were evaluated for their antioxidant effects. Compound **397** was more potent in terms of its DPPH radical effect (IC_50_: 25.0 μM) and this activity was higher than the standard gallic acid. On the other hand, compounds **398** (IC_50_: 322.4.0 μM) and **399** (IC_50_: 231.2 μM) exhibit quite low antioxidant activity [124].

**Figure 47:**
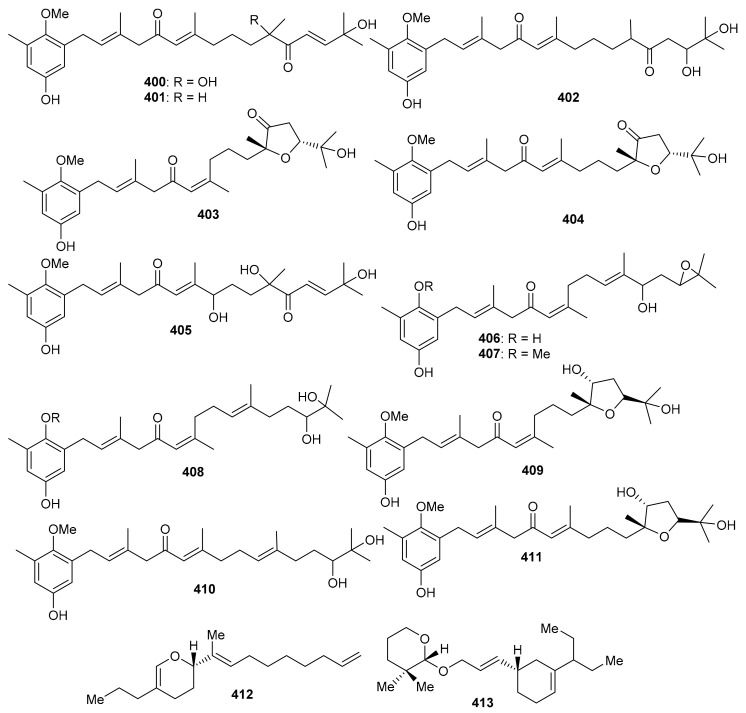
Structures of miscellaneous meroterpenoids **400**–**413**.

*Cystoseira usneoides* produces the meroterpenoids; cystodiones G–J (**400**–**403**), L (**404**) and M (**405**) along with cystones A–F (**406**–**411**) (Figure 47) [125]. All these compounds featured the toluhydroquinone core attached to a diterpenoid moiety. Compounds **400**–**411** illustrated radical-scavenging effects which ranged from 37% to 87%. Among these compounds, cystodiones G (**400**; 81%) and H (**401**; 77%) were the most potent. Moreover, cystodione G (**400**) and cystodione M (**405**) significantly inhibited the TNF-α production with 81% and 79% respectively while cystone C (**408**) demonstrated a moderate inhibition of 59% [125]. 2-[(*E*)-Deca-1,8-dien-10-yl]-11,12-dihydro-13-propyl-2*H*-pyran (**412**) and 1′-[(10*E*)-10-{10-pentan-4-yl}-cyclohex-4-enyl]-allyloxy)-tetrahydro-2′, 2′-dimethyl-2*H*-pyran (**413**) (Figure 47) were reported to be isolated from an animal source viz., *Paphia malabarica* [126]. In the DPPH antioxidant assay, compound **413** was slightly more potent (IC_50_ = 0.76 mg/mL) than compound **412** (IC_50_ = 0.78 mg/mL), while in the ABTS evaluation, compound **412** (IC_50_ = 0.92 mg/mL) was slightly more active than compound **413** (IC_50_ = 0.96 mg/mL). In addition both compounds were active towards COX-1 and COX-2 (IC_50_: 0.92 to 1.07 mg/mL) along with selectivity indices of ~1.1 mg/mL. Moreover compounds **412** (IC_50_ = 1.02 mg/mL) and **413** (IC_50_ = 1.06 mg/mL) illustrated 5-LOX inhibition effects [126].

**Figure 48:**
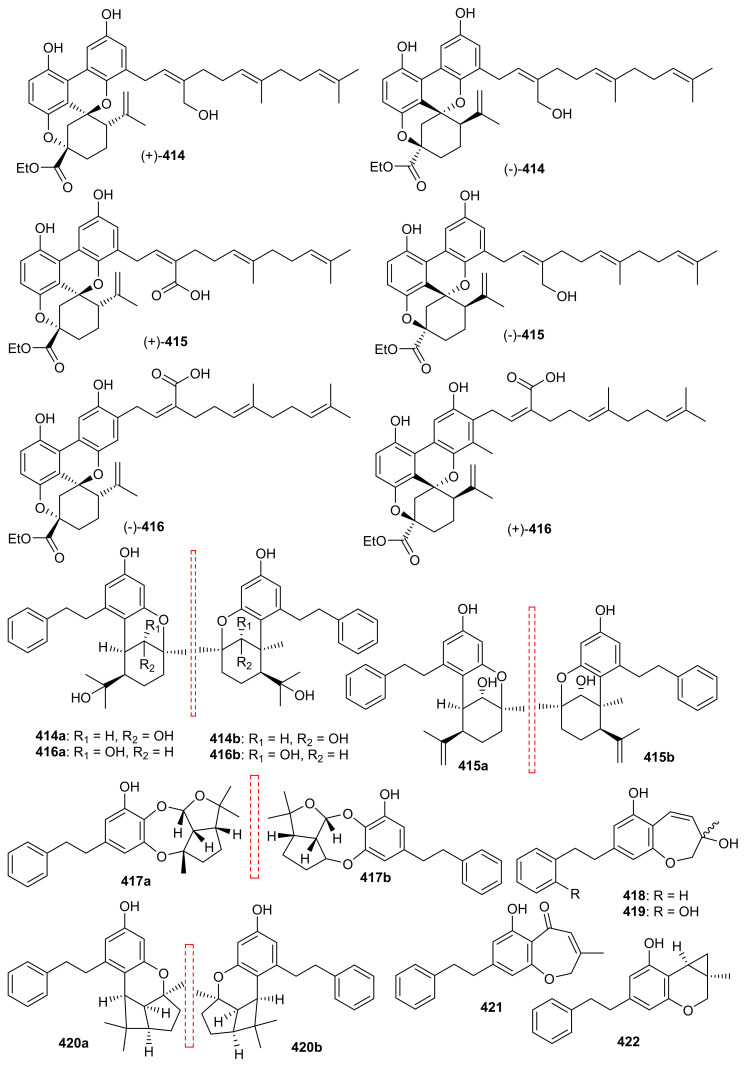
Structures of miscellaneous meroterpenoids **414**–**422**.

The meroterpenoid enantiomers, (±)-rasumatranin A–D (**414**–**417**) and (±)-radulanin M (**418**) and N (**419**) along with meroterpenoids **420**, **421** and (±)-radulanin I (**422**) (Figure 48) were isolated from *Radula sumatrana* [127]. Compounds **414**–**417** and **421** (Figure 48) are monoterpene-bibenzyl hybrid metabolites while **421** and **422** are hemiterpenoid-bibenzyl hybrid compounds. Among these compounds, **421** proved to be very potent towards MCF-7 (IC_50_: 3.8 µM), PC-3 (IC_50_: 6.6 µM) and SMMC-7721 7 (IC_50_: 3.5 µM) while **422** was only moderatively active towards these cancer cells with IC_50_ values 13.9–19.5 µM. On the other hand compounds **415** and **422** demonstrated moderate effects towards MCF-7 with IC_50_: 38.3 and 24.6 µM respectively [127].

**Figure 49:**
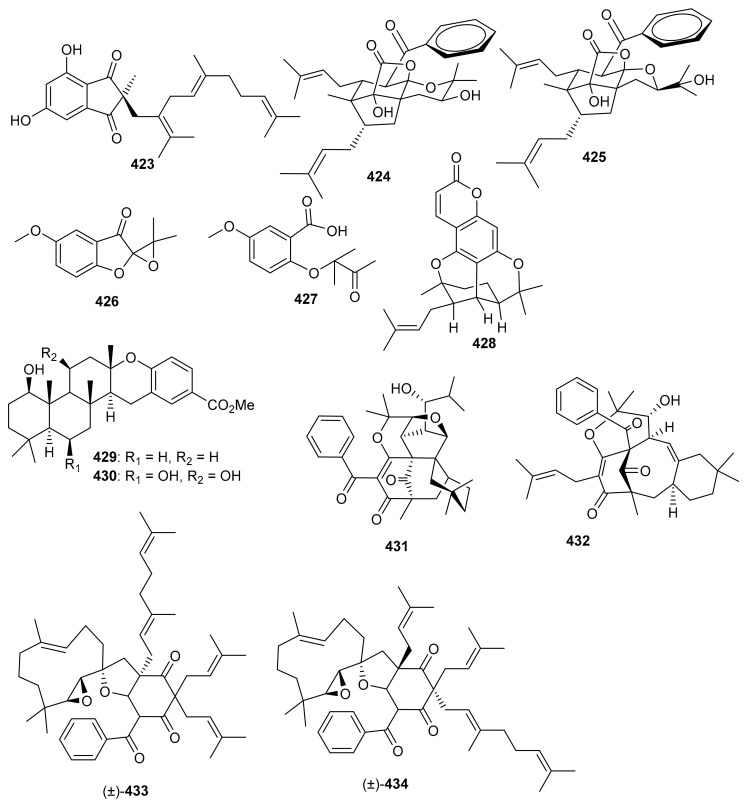
Structures of miscellaneous meroterpenoids **423**–**434**.

Meroindenon (**423**) was isolated from Streptomyces and illustrated moderate antimicrobial effects towards *B. subtilis*, *K. rhizophila*, and *S. aureus* [121]. Hyperinoids A (**424**) and B (**425**) (Figure 49) were isolated from *Hypericum patulum* and both compounds significantly inhibited NF-kB in RAW 246.7 macrophages [128]. Meroterpenoids **426** and **427** were produced by the mushroom *Panus lecomtei* [129] and prenylbruceol A was isolated from *Philotheca myoporoides* [130]. Moreover 6-deoxytolypodiol (**428**) and 11-hydroxytolypodiol (**429**) were produced by cyanobacterial sources. Only compound **430** displayed potent anti-inflammatory effects with IC_50_: 0.1 µM while the same compound possessed similar levels of TXB_2_ inhibitory effects as NSAID flurbiprofen [131]. Hyperprins A (**431**) and B (**432**) were produced by *Hypericum przewalskii* and both compounds featured a 6/6/6/6/5/5 hexacyclic core and a 6/8/6/6 tetracyclic system respectively [132]. In another report hypulatones A (**433**) and B (**434**) (Figure 49) were produced by *Hypericum patulum* and the latter compound remarkably inhibited the late current of Na_v_1.5 with IC_50_: 0.2 μM) [133].

**Figure 50:**
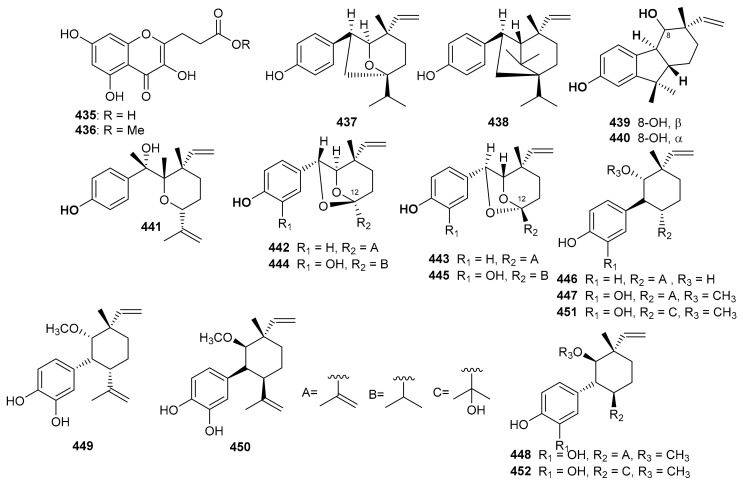
Structures of miscellaneous meroterpenoids **435**–**452**.

Ampechromonol A (**435**) and B (**436**) (Figure 50) were produced by *Ampelopsis cantoniensis* and displayed weak cytotoxic effects towards MCF-7 cells [134]. *Psoralea corylifolia* seed produced 7*β*, 13*β*-psoracorylifol B (**437**) and 7*β*, 8*α*-psoracorylifol D (**438**). Moreover, compounds **435**–**439** displayed moderate inhibitory effects towards DGAT1 with IC_50_: 67.1 and 99.5 μM, respectively. On the other hand, both compounds **435** and **436** illustrated weak inhibition towards DGAT2 with IC_50_: 132.9 and 134.2 μM, respectively [135]. In another report, corypsoriols A–N (**439**–**452**) (Figure 50) were isolated from *Psoralea corylifolia* [136].

**Table 5:** Sources and biological effects of meroterpenoids **373**–**422**.

Compounds	Source	Activities	Ref.
Magterpenoid C (**373**)	*Magnolia officinalis*	**Enzyme Inhibition**: PTP1B = IC_50_ 0.81 μM	[116]
Arnebinone B (**374**)	*Arnebia euchroma*	**Cytotoxic effects**: HepG2, SMMC-7721, QGY-7703 and HepG2/ADM IC_50_ ranging from 9.6 to 18.7 μM	[117]
Compound **375**	*Arnebia euchroma*	**Cytotoxic effects**: HepG2, SMMC-7721, QGY-7703 and HepG2/ADM IC_50_ ranging from 3.43 to 11.31 μM	[117]
Toluquinol-derivative (**378**)	*Carteriospongia* sp.	**Cytotoxic effects**: Molt 4 = IC_50_ 0.34 μg/mL; HL60 = IC_50_ 0.70 μg/mL; lymphoma U937 = IC_50_ 0.65 μg/mL; Sup-T1 = IC_50_ 0.33 μg/mL; oral Ca9-22 = IC_50_ 0.97 μg/mL; Cal-27 = IC_50_ 0.51 μg/mL; breast T-47D = IC_50_ 1.06 μg/mL	[119]
Sargaquinoic acid (**379**)	*Sargassum serratifolium*	**Enzyme Inhibition**: AChE = IC_50_ 69.3 μM; BChE = IC_50_ 10.5 μM; BACE1 = IC_50_ 12.1 μM	[106]
Sargahydroquinoic acid (**384**)	*Sargassum serratifolium*	**Enzyme Inhibition**: AChE = IC_50_ 124.3 μM; BChE = IC_50_ 15.2 μM; BACE1 = IC_50_ 4.4 μM	[106]
Tetronasin (**385**)	*Streptomyces* sp.	**Cytotoxic effects**: HeLa cells = IC_50_ 0.23 μM	[115]
Magterpenoid A (**386**)	*Magnolia officinalis*	**Enzyme Inhibition**: PTP1B = IC_50_ 1.44 μM	[116]
Nyingchinoids A (**388a,b**)	*Rhododendron nyingchiense*	**Enzyme Inhibition**: PTP1B = IC_50_ 43.6 μM	[105]
Nyingchinoids B (**389a,b**)	*Rhododendron nyingchiense*	**Enzyme Inhibition**: PTP1B = IC_50_ 38.1 μM	[105]
Compound **390**	*Villorita cyprinoides*	**Antioxidant effects**: DPPH = IC_50_ 0.59 mg/mL; ABTS = IC_50_ 0.65 mg/mL; **Enzyme Inhibition**: COX-1 = IC_50_ 0.94 mg/mL; COX-2 = IC_50_ 0.70 mg/mL	[122]
Compound **391**	*Villorita cyprinoides*	**Antioxidant effects**: DPPH = IC_50_ 0.54 mg/mL; ABTS = IC_50_ 0.62 mg/mL; **Enzyme Inhibition**: COX-1 = IC_50_ 0.86 mg/mL; COX-2 = IC_50_ 0.65 mg/mL	[122]
Compound **392**	*Villorita cyprinoides*	**Antioxidant effects**: DPPH = IC_50_ 0.69 mg/mL; ABTS = IC_50_ 0.64 mg/mL; **Enzyme Inhibition**: COX-1 = IC_50_ 0.91 mg/mL; COX-2 = IC_50_ 0.74 mg/mL	[122]
{Tetrahydro-3-methoxy-5-((*E*)-8,12-dimethyloct-8-enyl)-pyran-2-one (**393**)	*Villorita cyprinoides*	**Antioxidant effects**: DPPH = IC_50_ 0.70 mg/mL; ABTS = IC_50_ 0.76 mg/mL; Fe^2+^ = IC_50_ 0.83 mg/mL; H_2_O_2_ = IC_50_ 0.85 mg/mL; COX-1 = IC_50_ 0.99 mg/mL; COX-2 = IC_50_ 0.89 mg/mL	[123]
Dihydro-5-(8-(9,12-dihydro-8-methyl-11-propyl-2*H*-pyran-8-yl)-ethyl)-furan-2(3*H*)-one (**394**)}	*Villorita cyprinoides*	**Antioxidant effects**: DPPH = IC_50_ 0.63 mg/mL; ABTS = IC_50_ 0.79 mg/mL; Fe^2+^ = IC_50_ 0.83 mg/mL; H_2_O_2_ = IC_50_ 0.84 mg/mL; **Enzyme Inhibition**: COX-1 = IC_50_ 0.96 mg/mL; COX-2 = IC_50_ 0.84 mg/mL	[123]
Hexahydro-iso-chromenyl-meroterpenoid (**395**)	*Villorita cyprinoides*	**Antioxidant effects**: DPPH = IC_50_ 0.76 mg/mL; ABTS = IC_50_ 0.82 mg/mL; Fe^2+^ = IC_50_ 0.90 mg/mL; H_2_O_2_ = IC_50_ 0.86 mg/mL; **Enzyme Inhibition**: COX-1 = IC_50_ 1.05 mg/mL; COX-2 = IC_50_ 0.90 mg/mL	[123]
Hexahydro-iso-chromenyl-meroterpenoid (**396**)	*Villorita cyprinoides*	**Antioxidant effects**: DPPH = IC_50_ 0.79 mg/mL; ABTS = IC_50_ 0.81 mg/mL; Fe^2+^ = IC_50_ 0.89 mg/mL; H_2_O_2_ = IC_50_ 0.87 mg/mL; **Enzyme Inhibition**: COX-1 = IC_50_ 1.09 mg/mL; COX-2 = IC_50_ 0.89 mg/mL	[123]
2-(Tetrahydro-5-(4-hydroxyphenyl)-4-pentylfuran-3-yl)-ethyl-4-hydroxybenzoate (**397**)	*Hypnea musciformis*	**Antioxidant effects**: DPPH = IC_50_ 25.05 μM; Fe^2+^ ion chelating = IC_50_ 350.66 μM	[124]
2-2-[(4-Hydroxybenzoyl)-oxy]-ethyl-4-methoxy-4-2-[(4-methylpentyl)oxy]-3,4-dihydro-2*H*-6-pyranylbutanoic acid (**398**)	*Hypnea musciformis*	**Antioxidant effects**: DPPH = IC_50_ 322.4 μM; Fe^2+^ ion chelating = IC_50_ 5115.3 μM	[124]
3-((5-Butyl-3-methyl-5,6-dihydro-2*H*-pyran-2-yl)-methyl)-4-meth oxy-4-oxobutyl benzoate (**399**)	*Hypnea musciformis*	**Antioxidant effects**: DPPH = IC_50_ 231.2 μM Fe^2+^ ion chelating = IC_50_ 667.9 μM	[124]
2-((*E*)-deca-1,8-dien-10-yl)-11,12-dihydro-13-propyl-2H-pyran (**412**)	*Paphia malabarica*	**Antioxidant effects**: DPPH = IC_50_ 0.78 mg/mL; ABTS = IC_50_ 0.92 mg/mL; **Enzyme Inhibition**: COX-1 = IC_50_ 1.07 mg/mL; COX-2 = IC_50_ 0.95 mg/mL; 5-LOX = IC_50_ 1.02 mg/mL	[126]
1′-((10*E*)-10-(10-(pentan-4-yl)-cyclohex-4-enyl)-allyloxy)-tetrahydro-2′, 2′-dimethyl-2*H*-pyran (**413**)	*Paphia malabarica*	**Antioxidant effects**: DPPH = IC_50_ 0.76 mg/mL; ABTS = IC_50_ 0.96 mg/mL; **Enzyme Inhibition**: COX-1 = IC_50_ 1.05 mg/mL; COX-2 = IC_50_ 0.92 mg/mL; 5-LOX = IC_50_ 1.06 mg/mL	[126]
(±)-Rasumatranin B (**415**)	*Radula sumatrana*	**Cytotoxic effects**: MCF-7 = IC_50_: 38.3 µM	[127]
Compound **421**	*Radula sumatrana*	**Cytotoxic effects**: MCF-7: IC_50_: 3.8 µM; PC-3: IC_50_: 6.6 µM; SMMC-7721 7: IC_50_: 3.5 µM	[127]
(±)-Radulanin I (**422**)	*Radula sumatrana*	**Cytotoxic effects**: MCF-7 = IC_50_: 24.6 µM	[127]

## 10. Conclusions

In this review, the structures, chemical diversity, and biological properties of 452 new meroterpenoids have been reported. The chemical structures of meroterpenoids are extremely diverse, as may be noted by the various biosynthetic pathways and clearly demonstrated nature’s sophisticated synthetic protocols to generate this tremendous chemical diversity via simple and achiral starting units. As comprehensively explored in each section, these types of secondary metabolites possess a tremendous structural diversity resulting from such reactions as condensation, alkylation, oxidation, and reduction. Moreover, meroterpenoids incorporated multiple prenyl moieties or very complex ring cores, which furnish abundant molecular scaffolds for such a wide range of biological activities.

Due to their structural diversity, meroterpenoids illustrated a wide range of biological and pharmacological activities, viz., antimicrobial, anticancer/cytotoxic, antioxidant, anti-inflammatory, antiviral immunosuppressive, and anti-Leishmania. Moreover, these compounds are also reported to possess important enzyme inhibitory effects, viz., acetylcholinesterase, protein tyrosine phosphatase 1B (PTP1B), BACE1, dehydrogenase 1 (IDH1), α-glucosidase, influenza neuraminidase, Janus Kinase 3 (JAK3), HMG-CoA, aldose reductase, maltase, and β-hexosaminidase. Among phloroglucinol-derived metroterpenoids, psiguajavadial B (**2**), which was isolated from *Psidium guajava*, illustrated potent cytotoxic effects towards lung cancer (A549) with IC_50_ 0.90 µM. Moreover, eugenial C (**18**), which was produced by *Eugenia umbelliflora*, possessed significant cytotoxic effects towards leukemia cells (K562) with IC_50_ 0.38 μM. On the other hand, *Eucalyptus robusta* produced eucarobustols A (**63**), G (**69**), and I (**71**) and these compounds illustrated significant PTP1B inhibition (antidiabetic effects) with IC_50_ 1.3, 1.8, and 1.6 μM, respectively. Among syncarpic acid/β-triketones-based meroterpenes, frutescones O (**182**) exhibited anti-inflammatory activity with an IC_50_ value of 0.36 μM while hyperjaponols B (**195a**) D (**197**) demonstrated potent antiviral effects towards the EBV virus with EC_50_ 0.57 and 0.49 μM respectively.

Marinocyanin A (**240**) is an alkaloidal based meroterpenoid and is isolated from *Actinomycete* strains; it demonstrates potent cytotoxic effects towards colon cancer (HCT-116) with IC_50_ 0.049 μM. Furthermore, this compound also possesses significant antimicrobial effects towards *Candida albicans* (MIC: 0.95 μM), and *Staphylococcus aureus* (MIC: 2.3 μM). Similarly, another alkaloidal based meroterpenoid marinocyanin B (**241**) also demonstrated potent cytotoxic effects towards HCT-116 with IC_50_ 0.029 μM. Among sesquiterpene-based meroterpenoids, chartarolide A (**274**) was produced by *Niphates recondite* and demonstrated significant cytotoxic effects towards HCT-116, HepG2, BGC-823, A2780, and MCF7 with IC_50_ 1.9, 1.8, 1.3, 1.5, and 1.4 μM, respectively. Magterpenoid C (**373**) demonstrated potent antidiabetic effects (PTP1B = IC_50_ 0.81 μM). Notably meroterpene **378** possessed potent cytotoxic effects towards Molt 4, HL60, lymphoma U937, Sup-T1, oral Ca9-22, and with IC_50_ < 1.0 μg/mL. In addition, tetronasin (**385**) which was produced by *Streptomyces* sp., illustrated significant cytotoxic effects towards HeLa cells with IC_50_ 0.23 μM. From the large library of bioactive meroterpenoids described in this detailed review, we hope a reasonable range of new lead structures may enter into the drug development process to treat debillitating diseases in the future.

## Figures and Tables

**Table 3:** Alkaloids-based meroterpenoids.

Compounds	Source	Anticancer	Ref.
Marinocyanin A (**240**)	*Actinomycete* strains	**Cytotoxic effects**: HCT-116 = IC_50_ 0.049 μM; **Antimicrobial effects**: *Candida albicans* = MIC 0.95 μM; *Staphylococcus aureus* = MIC 2.3 μM	[79]
Marinocyanin B (**241**)	*Actinomycete* strains	**Cytotoxic effects**: HCT-116 = IC_50_ 0.029 μM; **Antimicrobial effects**: *Candida albicans* = MIC 5.79 μM; *Staphylococcus aureus* = MIC 33.92 μM	[79]
Marinocyanin C (**242**)	*Actinomycete* strains	**Antimicrobial effects**: *Candida albicans* = MIC 3.90 μM; *Staphylococcus aureus* = MIC 30.71 μM	[79]
Marinocyanin D (**243**)	*Actinomycete* strains	**Antimicrobial effects**: *Candida albicans* = MIC 14.65 μM; *Staphylococcus aureus* = MIC 36.62 μM	[79]
Marinocyanin E (**244**)	*Actinomycete* strains	**Antimicrobial effects**: *Candida albicans* = MIC 14.65 μM; *Staphylococcus aureus* = MIC 36.62 μM	[79]
Marinocyanin F (**245**)	*Actinomycete* strains	**Antimicrobial effects**: *Candida albicans* = MIC 14.65 μM; *Staphylococcus aureus* = MIC 36.62 μM	[79]
Lavanducyanin (**246**)	*Streptomyces* sp.	**Antimicrobial effects**: *Candida albicans* = MIC 114.67 μM; *Staphylococcus aureus* = MIC 56.93 μM	[79]

**Table 4:** Sesquiterpene-based, chromane/chromene and flavone derived meroterpenoids.

Compounds	Source	Activities	Ref.
Langcoquinone A (**248**)	*Spongia* sp.	**Antimicrobial effects**: *Staphylococcus aureus* = MIC 12.5 μM; *Bacillus subtilis* = MIC 12.5 μM	[82]
Langcoquinone B (**249**)	*Spongia* sp.	**Antimicrobial effects**: *Staphylococcus aureus* = MIC 12.5 μM; *Bacillus subtilis* = MIC 12.5 μM	[82]
Langconol A (**250**)		**Antimicrobial effects**: *B. subtilis* MIC 12.5 μM	[83]
Langconol C (**252**)		**Antimicrobial effects**: *B. subtilis* = MIC 25.0 μM	[83]
Langcoquinone C (**253**)		**Antimicrobial effects**: *Staphylococcus aureus* = MIC 12.50 μM; *Bacillus subtilis* = MIC 6.25 μM	[83]
Aminoquinone (**257**)	*Dysidea* sp.	**Antimicrobial effects**: *B. subtilis* = MIC 50.0 μg/mL; *S. aureus* = MIC 50.0 μg/mL; *E. coli* = MIC 50.0 μg/mL	[84]
Nakijinol G (**267**)	*Hyrtios* sp.	**Enzyme Inhibition**: PTP1B = IC_50_ 4.8 μM	[87]
Dysivillosin A (**270**)	*Dysidea villosa*	**Enzyme Inhibition**: *β*-hexosaminidase = IC_50_ 8.2 μM	[88]
Dysivillosin B (**271**)		**Enzyme Inhibition**: *β*-hexosaminidase = IC_50_ 10.2 μM	[88]
Dysivillosin C (**272**)		**Enzyme Inhibition**: *β*-hexosaminidase = IC_50_ 19.9 μM	[88]
Dysivillosin D (**273**)		**Enzyme Inhibition**: *β*-hexosaminidase = IC_50_ 16.2 μM	[88]
Chartarolide A (**274**)	*Niphates recondite*	**Cytotoxic effects**: HCT-116 = IC_50_ 1.9 μM; HepG2 = IC_50_ 1.8 μM; BGC-823 = IC_50_ 1.3 μM; NCI-H1650 = IC_50_ 5.5 μM; A2780 = IC_50_ 1.5 μM; MCF7 = IC_50_ 1.4 μM	[90]
Chartarolide B (**275**)	*Niphates recondite*	**Cytotoxic effects**: HCT-116 = IC_50_ 2.3 μM; HepG2 = IC_50_ 2.8 μM; BGC-823 = IC_50_ 1.6 μM; NCI-H1650 = IC_50_ 4.8 μM; A2780 = IC_50_ 3.2 μM; MCF7 = IC_50_ 3.8 μM	[90]
Chartarolide C (**276**)	*Niphates recondite*	**Cytotoxic effects**: HCT-116 = IC_50_ 7.8 μM; HepG2 = IC_50_ 8.9 μM; BGC-823 = IC_50_ 5.4 μM; NCI-H1650 = IC_50_ 11.3 μM; A2780 = IC_50_ 12.5 μM; MCF7 = IC_50_ 8.7 μM	[90]
Terretonin N (**304**)	*Nocardiopsis* sp.	**Antimicrobial effects**: *S. warneri* = IZ 14 mm *E. coli* = IZ 8 mm	[98]
Rubiginosin A (**306**)	*Rhododendron rubiginosum*	**Cytotoxic effects**: A549 = IC_50_ 16.15 μM; HCT116 = IC_50_ 15.56 μM; SK-HEP-1 = IC_50_ 13.80 μM; HL-60 = IC_50_ 12.84 μM	[101]
Rubiginosin B (**307**)	*Rhododendron rubiginosum*	**Cytotoxic effects**: HCT116 = IC_50_ 65.72 μM; SK-HEP-1 = IC_50_ 84.66 μM	[101]
Rubiginosin C (**308**)	*Rhododendron rubiginosum*	**Cytotoxic effects**: A549 = IC_50_ 40.45 μM; HCT116 = IC_50_ 17.43 μM; SK-HEP-1 = IC_50_ 26.26 μM; HL-60 = IC_50_ 16.44 μM	[101]
Rubiginosin D (**309**)	*Rhododendron rubiginosum*	**Cytotoxic effects**: A549 = IC_50_ 49.18 μM; HCT116 = IC_50_ 32.17 μM; SK-HEP-1 = IC_50_ 13.66 μM; HL-60 = IC_50_ 40.07 μM	[101]
Rubiginosin E (**310**)	*Rhododendron rubiginosum*	**Cytotoxic effects**: A549 = IC_50_ 38.90 μM; HCT116 = IC_50_ 38.90 μM; SK-HEP-1 = IC_50_ 38.90 μM; HL-60 = IC_50_ 38.90 μM	[101]
Rubiginosin F (**311**)	*Rhododendron rubiginosum*	**Cytotoxic effects**: A549 = IC_50_ 38.90 μM; HCT116 = IC_50_ 38.90 μM; SK-HEP-1 = IC_50_ 38.90 μM; HL-60 = IC_50_ 38.90 μM	[101]
Rubiginosins G (**312**)	*Rhododendron rubiginosum*	**Cytotoxic effects**: A549 = IC_50_ 38.90 μM; HCT116 = IC_50_ 38.90 μM; SK-HEP-1 = IC_50_ 38.90 μM; HL-60 = IC_50_ 38.90 μM	[101]
Anthopogochromene A (**313**)	*Rhododendron rubiginosum*	**Cytotoxic effects**: A549 = IC_50_ 38.90 μM; HCT116 = IC_50_ 38.90 μM; SK-HEP-1 = IC_50_ 38.90 μM; HL-60 = IC_50_ 38.90 μM	[101]
Anthopogochromene B (**314**)	*Rhododendron rubiginosum*	**Cytotoxic effects**: A549 = IC_50_ 38.90 μM; HCT116 = IC_50_ 38.90 μM; SK-HEP-1 = IC_50_ 38.90 μM; HL-60 = IC_50_ 38.90 μM	[101]
Isopolycerasoidol (**315**)	*Sargassum siliquastrum*	Antioxidant effects: DPPH = EC_50_ 8.23 μM; ABTS = EC_50_ 2.33 μM	[103]
Sargachromanol D (**316**)	*Sargassum siliquastrum*	Antioxidant effects: DPPH = EC_50_ 26.35 μM; ABTS = EC_50_ 4.84 μM	[103]
Sargachromanol E (**317**)	*Sargassum siliquastrum*	Antioxidant effects: DPPH = EC_50_ 23.84 μM; ABTS = EC_50_ 4.57 μM	[103]
Sargachromanol G (**318**)	*Sargassum siliquastrum*	Antioxidant effects: DPPH = EC_50_ 33.43 μM; ABTS = EC_50_ 4.05 μM	[103]
Sargachromanol I (**319**)	*Sargassum siliquastrum*	Antioxidant effects: DPPH = EC_50_ 32.83 μM; ABTS = EC_50_ 6.86 μM	[103]
